# Bioinformatics Screen Reveals Gli-Mediated Hedgehog Signaling as an Associated Pathway to Poor Immune Infiltration of Dedifferentiated Liposarcoma

**DOI:** 10.3390/cancers15133360

**Published:** 2023-06-27

**Authors:** Erik P. Beadle, Natalie E. Bennett, Julie A. Rhoades

**Affiliations:** 1 Program in Cancer Biology, Vanderbilt University, Nashville, TN 37232, USA; 2 Department of Biomedical Engineering, Vanderbilt University, Nashville, TN 37232, USA; 3 Department of Medicine, Vanderbilt University Medical Center, Nashville, TN 37232, USA; 4 Division of Clinical Pharmacology, Vanderbilt University Medical Center, Nashville, TN 37232, USA; 5 Department of Veterans Affairs, Tennessee Valley Health Care, Nashville, TN 37212, USA

**Keywords:** liposarcoma, hedgehog signaling, differentiation, immune infiltration, microenvironment, Gli2

## Abstract

**Simple Summary:**

Liposarcomas (LPSs) are a rare soft tissue malignancy with a variable clinical prognosis dependent on tumor differentiation. Well-differentiated (WDLPS) and dedifferentiated (DDLPS) liposarcomas most commonly occur both in extremities and the retroperitoneal cavity. Surgical resection and post-operative monitoring are effective forms of treatment; however, many tumors do recur and require chemotherapeutic intervention. Liposarcomas have high intratumoral variability, which can influence drug response and microenvironment interactions. Using a broad bioinformatics approach, we show that Hedgehog signaling, a developmental pathway, is upregulated in DDLPS and correlates with a tumor gene expression signature that suggests reduced immune cell infiltration and increased extracellular matrix (ECM) protein expression.

**Abstract:**

Liposarcomas are the most diagnosed soft tissue sarcoma, with most cases consisting of well-differentiated (WDLPS) or dedifferentiated (DDLPS) histological subtypes. While both tumor subtypes can have clinical recurrence due to incomplete resections, DDLPS often has worse prognosis due to a higher likelihood of metastasis compared to its well-differentiated counterpart. Unfortunately, targeted therapeutic interventions have lagged in sarcoma oncology, making the need for molecular targeted therapies a promising future area of research for this family of malignancies. In this work, previously published data were analyzed to identify differential pathways that may contribute to the dedifferentiation process in liposarcoma. Interestingly, Gli-mediated Hedgehog signaling appeared to be enriched in dedifferentiated adipose progenitor cells and DDLPS tumors, and coincidentally Gli1 is often co-amplified with MDM2 and CDK4, given its genomic proximity along chromosome 12q13-12q15. However, we find that Gli2, but not Gli1, is differentially expressed between WDLPS and DDLPS, with a noticeable co-expression signature between Gli2 and genes involved in ECM remodeling. Additionally, Gli2 co-expression had a noticeable transcriptional signature that could suggest Gli-mediated Hedgehog signaling as an associated pathway contributing to poor immune infiltration in these tumors.

## 1. Introduction

Liposarcomas (LPSs) are soft tissue malignant neoplasms that have the greatest incidence amongst all soft tissue sarcomas [1,2,3]. The five histological subtypes of LPS consist of well-differentiated (WDLPS)/atypical lipomatous tumors (ALTs), dedifferentiated (DDLPS), myxoid, pleomorphic, and round cell, with the cell of origin and genetic abnormalities varying extensively across the different subtypes. WDLPS (~40%) and DDLPS (~18–20%) make up over 60% of LPS cases and share initiating chromosomal abnormalities that give rise to tumor development [4]. Importantly, WDLPS and DDLPS can originate in the retroperitoneal cavity, where surgical resection and post-operative follow-up are the first line of treatment. Clinical follow-up, particularly for WDLPS, is necessary due to the high incidence of recurrence and further exacerbates the need for a multidisciplinary team of practitioners, particularly with expertise in soft tissue sarcoma management [5]. One key complication that clinicians face is variable degrees of differentiation within LPS tumors. Intratumoral variability can drastically affect prognosis, recurrence, and likelihood of metastasis, making tumor differentiation a potential mechanism to target in liposarcoma development. In this study, we examined several previously published datasets through the Gene Expression Omnibus (GEO) [6] and CBioPortal [7,8] to identify potential targets that could be contributing to dedifferentiation during LPS tumor formation.

## 2. Results

### 2.1. Hedgehog Signaling Is Enriched in Dedifferentiated Adipose Progenitors and DDLPS Tumors

**Figure 1:**
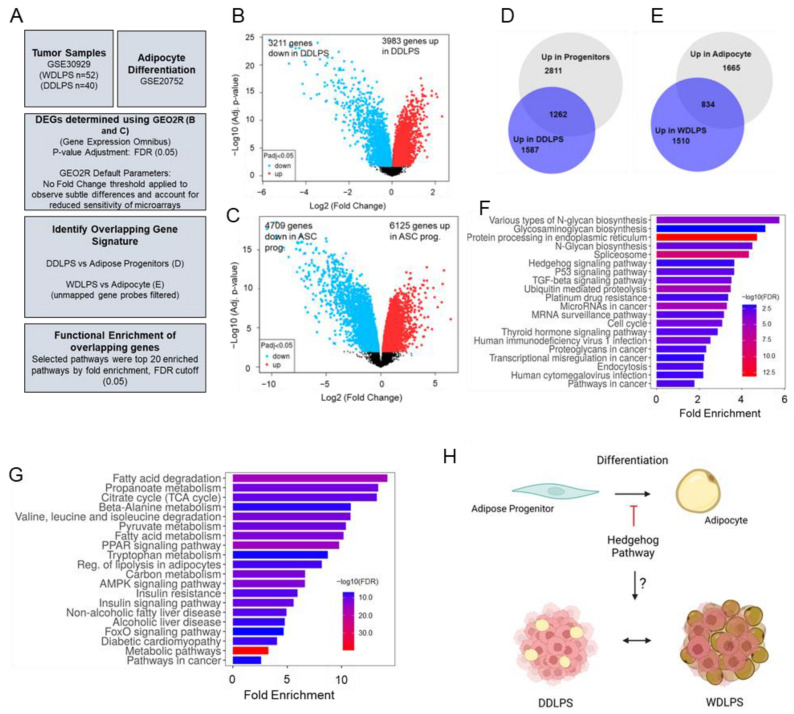
Comparative transcriptional analysis reveals enrichment of Hedgehog signaling in dedifferentiated adipose progenitors and dedifferentiated liposarcoma tumors. (**A**) Data accession and analysis pipeline. Tumor transcriptomics collected from GSE30929. Adipocyte differentiation transcriptomics collected from GSE20752. FDR *p*-value adjustment (0.05), no fold change threshold. (**B**) Differentially expressed genes between adipose progenitors and mature adipocytes. (**C**) Differentially expressed genes between WDLPS and DDLPS tumors. (**D**) Upregulated genes in DDLPS and Adipose progenitors. (**E**) Upregulated genes in WDLPS and adipocytes. (**F**) KEGG pathway analysis of overlapping upregulated genes in DDLPS and adipose progenitors. (**G**) KEGG pathway analysis of overlapping upregulated genes in WDLPS and adipocytes. Presented pathways were top 20 results based on fold enrichment, with an FDR cutoff of *p* < 0.05. (**H**) Hedgehog signaling canonically represses adipogenesis, but its overall role in the differentiation of liposarcoma is unknown.

Liposarcoma (LPS) tumors can vary extensively based on their grade of differentiation [1,2,9,10,11,12]. While there are five described subtypes of liposarcoma, we sought to better understand the underlying molecular differences between the well-differentiated (WDLPS) and dedifferentiated (DDLPS) subtypes that can cause varying degrees of tumor differentiation, which can be a contributing factor to clinical prognosis [3]. Using transcriptomics data collected from the Gene Expression Omnibus (GEO [6]) (Figure 1A), we evaluated differentially expressed genes (DEGs) between adipose progenitors and mature adipocytes (GSE20697 [13]) (Figure 1B), followed by DEGs upregulated in either WDLPS or DDLPS tumors (GSE30929 [14]) (Figure 1C). The inclusion criterion for genes in subsequent analyses was an adjusted *p*-value < 0.05, after Benjamini–Hochberg FDR correction. No fold change threshold was applied to observe subtle differences between groups, as well as accounting for reduced sensitivity of microarray platforms. Unmapped array probes were filtered from comparative analysis and functional enrichment classification (Figure 1A). The DEGs from the progenitor/adipocyte comparative analyses were then evaluated for overlap with their respective tumor tissue counterparts, based on differentiation status with adipose progenitors being compared to DDLPS tumors (Figure 1D) and adipocytes compared to WDLPS tumors (Figure 1E). Using BioVenn, we compared lists of mapped gene identifiers to identify genes that were commonly upregulated between progenitors and DDLPS or adipocytes and WDLPS [15]. In total, 1262 genes were found to be commonly upregulated in both adipose progenitors and DDLPS tumors (Figure 1D, Table S1), while 834 genes were preferentially expressed in adipocytes and WDLPS tumors (Figure 1E, Table S2).

The overlapping genes identified in Figure 1D,E were then input into a KEGG pathway analysis for functional enrichment classification. The top 20 pathways by fold enrichment and FDR cutoff (0.05) are presented in Figure 1F,G. Genes enriched in dedifferentiated adipose progenitor cells and DDLPS were functionally enriched for protein processing, proteoglycan biosynthesis, Hedgehog and TGF-B signaling, and stem cell pluripotency (Figure 1F). As expected, adipocytes and WDLPS tumors were preferentially enriched for fatty acid metabolism, PPAR signaling, AMPK signaling, and other metabolic pathways (Figure 1G). Interestingly, other works have previously shown that Hedgehog signaling actively represses adipogenic differentiation of mesenchymal progenitors [16,17,18,19,20,21], but its role in the dedifferentiation of liposarcoma tumors is largely unknown. Thus, we hypothesized that adipogenic repression by Hedgehog may drive the transition from mesenchymal progenitor biology to liposarcoma tumor phenotype (Figure 1H and Figure S1).

### 2.2. Chromosome 12q13-12q15 Amplification Increases Gli1 Expression, but Not Gli2

**Figure 2:**
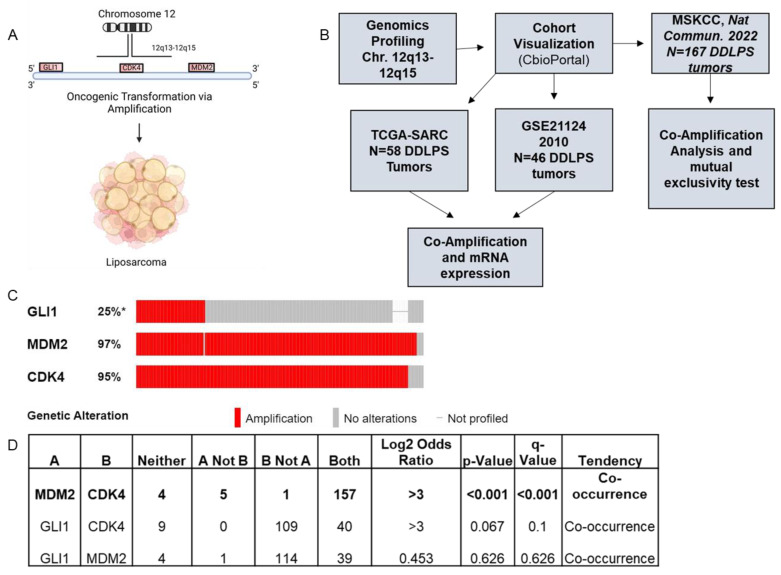
Gli1 amplification can occur with MDM2 and CDK4 amplification. (**A**) Oncogenic transformation of adipocytes and adipose progenitor cells is often driven by chromosome 12q13-15, which includes MDM2 and CDK4. Gli1, a Hedgehog transcription factor, shares this chromosome segment. (**B**) Genomics data acquisition and analysis pipeline. (**C**) Amplification distribution of Gli1, MDM2, and CDK4 in the MSKCC patient cohort (*n* = 167). (**D**) Mutual exclusivity test of co-amplifications. * *p* < 0.05.

**Figure 3:**
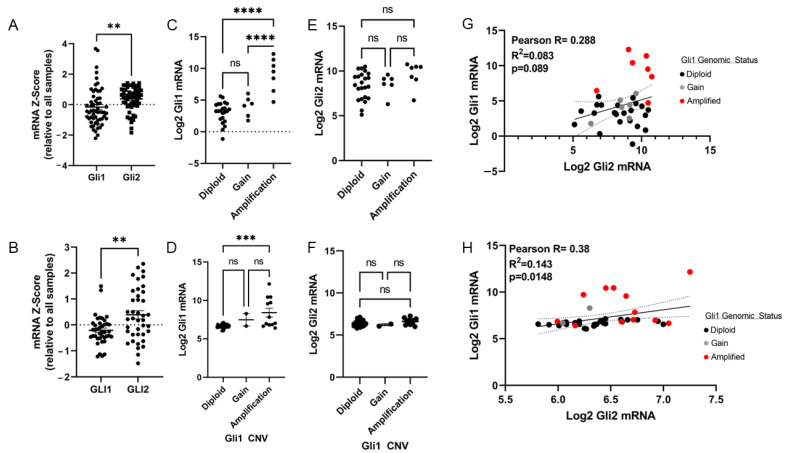
Gli2 has higher expression than Gli1 in DDLPS tumors but not as a result of Gli1 amplification. Gli1 and Gli2 mRNA expressions were evaluated in two separate cohorts (TCGA-SARC *n* = 58) (**A**) and GSE21124 (*n* = 50) of DDLPS tumors (**B**). Gli1 mRNA expression was compared to Gli1 genomic status in the respective cohorts (**C**,**D**), as was Gli2 mRNA expression (**E**,**F**). Pearson correlation analysis was performed to evaluate the strength of the relationship between Gli1 and Gli2 expression in DDLPS tumors in both cohorts (**G**,**H**). Statistical evaluation was performed following Rout’s outlier test with subsequent Student’s *t*-test or one-way ANOVA when appropriate. Outliers were removed as follows: 3B, Gli1 *n* = 6; 3C, Diploid *n* = 7, Gain *n* = 2, Amp. *n* = 1; 3D, Diploid, *n* = 1. Paired values from patient tumors were removed following outlier removal. (ns: not significant, ** *p* < 0.01, *** *p* < 0.001, **** *p* < 0.0001).

Liposarcoma tumors have many chromosomal amplifications that result in oncogenic transformation, with the most notable being the amplification of chromosome 12q13-12q15. Both WDLPS and DDLPS often contain this chromosomal amplification which can consist of a dual amplification of MDM2 (Mouse Double Minute 2 Homolog) and CDK4 (Cyclin-Dependent Kinase 4), in addition to Sarcoma Amplified Sequence (SAS/TSPAN31) and HMGA2 (High-Mobility Group AT-Hook 2) [9]. Previous papers have mapped the Gli1 genomic locus to the same chromosome arm near 12q13.3 [22,23,24,25] (Figure 2A), leading us to hypothesize that Gli1 amplification may be a driving factor behind the upregulation of Hedgehog signaling in DDLPS tumors. Using CBioPortal [7,8], we accessed three independent patient cohorts from previous publications to evaluate Chromosome 12q13-12q15 amplification status, gene expression, and amplification co-occurrence (Figure 2B). Gli1 displayed amplification in 25% of the MSKCC-IMPACT 2022 cohort [26] (*n* = 167) (Figure 2C) as well as 14% of the TCGA-SARC [12] cohort (*n* = 59) and ~24% of the GSE21124 [27] cohort (*n* = 50) (Figure S2A,B). A mutual exclusivity test was performed in CBioPortal [7,8] to evaluate co-amplification between Gli1, CDK4, and MDM2. As expected, CDK4 and MDM2 significantly co-occurred with one another in all three cohorts (Figure 2D and Figure S2A,B). Gli1 was more frequently co-amplified with CDK4, likely due to the genomic locus proximity of these two genes (Figure 2A), albeit not significant in either the MSKCC 2022 [26] cohort or TCGA-SARC [12] cohort (Figure 3D and Figure S2A). We next sought to evaluate if chromosome 12q13-12q15 amplification resulted in a concomitant increase in Gli1 mRNA expression. It was determined that Gli1 expression did not increase with MDM2 or CDK4 amplification in either the TCGA-SARC [12] or GSE21124 [27] cohorts. mRNA expression values were not profiled in the MSK-IMPACT patient cohort, so only genomic profiling could be performed on this dataset (Figure S1C,D). Gli1 displays a genomic association with LPS tumor initiation; however, it is not the only transcriptional activator of the Hedgehog pathway and can often display both overlapping and distinct transcriptional targets with that of Gli2 [28,29], the other primary Hedgehog transcriptional activator.

Both Gli1 and Gli2 were profiled to determine mRNA expression levels, as well as if Gli2 mRNA expression could also be increased by Gli1 amplification. Using the TCGA-SARC [12] (*n* = 58) cohort and GSE21124 [27] (*n* = 46) cohort, it was determined that on average, Gli2 expression was significantly higher in DDLPS tumors except for statistical outliers removed by Rout’s outlier test (Figure 3A,B). As expected, Gli1 mRNA expression was increased by Gli1 chromosomal amplification (Figure 3C,D), whereas Gli1 amplification had no bearing on Gli2 gene expression, suggesting that Gli1 amplification does not increase Gli2 expression in DDLPS tumors. Pearson correlation analysis revealed a modest positive correlation between Gli1 and Gli2 gene expression, although the amplification status of Gli1 had no bearing on this relationship (Figure 3G,H).

### 2.3. Gli2 Expression and Downstream Hedgehog Signaling Are Elevated in DDLPS Tumors

**Figure 4:**
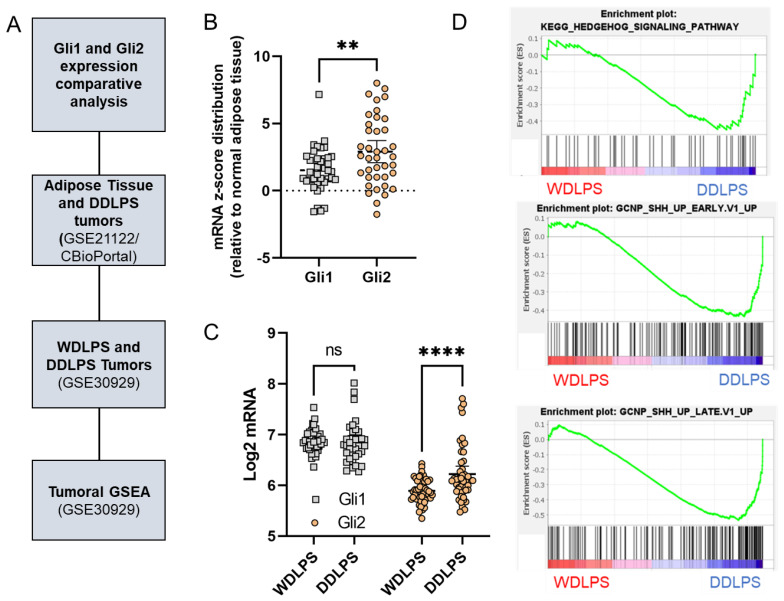
Gli2 expression and downstream Hedgehog signaling are elevated in DDLPS tumors. Gli1 and Gli2 mRNA expressions were evaluated in two separate cohorts (GSE21122 *n* = 9 adipose tissue, *n* = 46 DDLPS) and GSE30929 (*n* = 52 WDLPS, *n* = 40 DDLPS) (**A**). Gli1 and Gli2 expression levels were profiled in 46 DDLPS tumors relative to normal fat samples (*n* = 9). mRNA z-scores were extracted from CBioportal and outlier corrected using Rout’s outlier test. Seven Gli1 expression outliers were identified, and the corresponding Gli2 expression values were removed from the analysis (**B**). Gli1 and Gli2 expression levels were evaluated to determine expression levels between WDLPS (*n* = 52) and DDLPS (*n* = 40) tumors. Six Gli1 outliers were removed as well as their corresponding Gli2 expression values. Significance was determined using two-way ANOVA with Sidak’s test for multiple comparisons (**C**). GSEA of expression files collected from GSE30929 cohort (**D**). (ns: not significant, ** *p* < 0.01, **** *p* < 0.0001).

Based on the high variability of Gli1/2 expression in DDLPS tumors, we sought to better characterize their expression profiles relative to normal adipose tissue and WDLPS tumors. Using the tumor cohorts identified in Figure 4A, Gli1 and Gli2 expressions were evaluated and compared (GSE21122 [27]: *n* = 9 normal adipose tissue, *n* = 46 DDLPS) (GSE30929 [14]: *n* = 52 WDLPS, *n* = 40 DDLPS). Interestingly, we found that Gli2 mRNA z-score distribution was significantly higher than that of Gli1 in DDLPS tumors relative to normal adipose tissue (Figure 4B). Additionally, while Gli1 expression was slightly elevated when compared to Gli2 in both WDLPS and DDLPS tumors in the GSE30929 [14] cohort, no significant difference in Gli1 expression was present when comparing the two separate tumor types (Figure 4C).

However, we observed a significant increase in Gli2 expression in DDLPS tumors compared to WDLPS tumors (Figure 4C), leading us to hypothesize that Gli2 may play a greater functional role in tumor dedifferentiation compared to Gli1. Gene Set Enrichment Analysis (GSEA) was performed on the tumors from the GSE30929 [14] cohort (WDLPS and DDLPS) to determine if downstream Hedgehog target genes were also enriched in DDLPS tumors. Using adipogenesis and adipocytokine signaling (Figure S3) as validation gene sets, we observed a moderate enrichment of Hedgehog signaling components and both early and late downstream Hedgehog signaling (Figure 4D).

### 2.4. Gli2-Focused Co-Expression Analysis Reveals an Inverse Relationship between Gli2 and Immune Cell Population Markers While Establishing a Positive Correlation to Fibroblasts and ECM Gene Markers

The previous results provided rationale that Gli2 may contribute a larger role in the state of dedifferentiation for liposarcoma tumors than Gli1. Given the low incidence of liposarcoma tumors, research has lagged, leading to a shortfall in sequencing datasets readily available. Thus, we collected co-expression data on bulk tumor RNA-seq collected for the TCGA-SARC [12] cohort through CBioPortal [7,8] to better understand transcriptional associations that Gli2 may display within DDLPS tumors. Additionally, identical analysis was performed on the GSE21124 [27] cohort. However, this cohort used microarrays to evaluate tumoral gene expression, resulting in less sensitivity when performing co-expression analyses.

**Figure 5:**
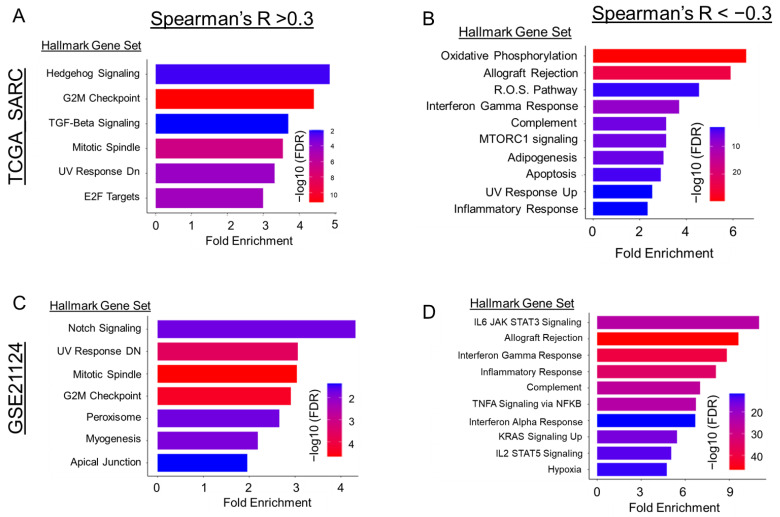
Gene ontology and functional enrichment of Gli2 co-expressed genes. Using CBioPortal, two separate DDLPS cohorts were analyzed using co-expression analysis relative to Gli2. Genes were filtered to have a Spearman’s R of +/− 0.3 as well as q-value < 0.05. Gene lists were input and subsequently analyzed for enrichment across both cohorts. Genes with an R > +0.3 were preferentially enriched for cell cycle and signaling pathways (**A**,**C**). Genes with an R < −0.3 were preferentially enriched for inflammatory signaling (**B**,**D**). Top 10 pathways were selected based on fold enrichment and FDR cutoff (0.05).

Using CBioPortal [7,8], transcriptional co-expression analysis was performed to discern stronger relationships that Gli2 may functionally be involved in within DDLPS tumors. The inclusion criteria for enrichment analyses included correlated genes that had both statistical significance with a q-value of <0.05, following false discovery correction, in addition to a cutoff for Spearman’s R of at least +/− 0.3 to identify transcriptional relationships with a modest correlation with Gli2 expression. As anticipated, Hedgehog signaling had the greatest fold enrichment with Gli2-associated co-expression, closely followed by cell-cycle-associated gene sets, TGF-Beta signaling, and UV damage response (Figure 5A). In converse, we observed a strong inverse relationship between Gli2 and pathways involved in oxidative phosphorylation, inflammatory responses, adipogenesis, and apoptosis (Figure 5B). Upon validation in the secondary cohort (GSE21124 [27]), we observed Notch signaling enrichment instead of Hedgehog signaling and TGF-B signaling, in addition to cell-cycle-related pathways, myogenesis, and UV response (Figure 5C). Developmental pathways have frequently been shown to crosstalk, so these results were not unexpected. However, we found that inflammatory signaling and interferon responses were largely retained with an inverse association with Gli2 expression (Figure 5D). Using the Human Protein Atlas [30,31,32], we evaluated Gli2 expression within normal subcutaneous adipose tissue [33,34] and found that Gli2 expression was limited to mesenchymal cell populations, such as fibroblasts and adipose progenitors, while absent in most immune cell populations (Figure S4), leading us to hypothesize that Hedgehog signaling may facilitate stromal interactions within the tumor microenvironment. Furthermore, Gli2 displayed co-expression with genes functionally annotated as ECM remodeling or ECM organization within fibroblasts and connective tissue cells (Figures S5 and S6). Interestingly, many of these genes identified in both bulk [33] and single-cell RNA sequencing [34] displayed functional enrichment for regulatory mechanisms in mesenchymal lineage differentiation, with a noted negative regulatory mechanism in adipocyte differentiation (Figure S6). Taken together, these results suggested that Gli2 activity could indeed vary based on the degree of differentiation with liposarcoma tumors, albeit a limited conclusion due to lack of robust tumor sequencing data at this time.

To further clarify the functional enrichment of the Gli2 co-expression network within DDLPS tumors, we identified genes that displayed the strongest correlations with Gli2 expression across the three independent patient cohorts (+/− 0.5–0.99) (TCGA-SARC [12], GSE21124 [27], GSE30929 [14]).

**Figure 6:**
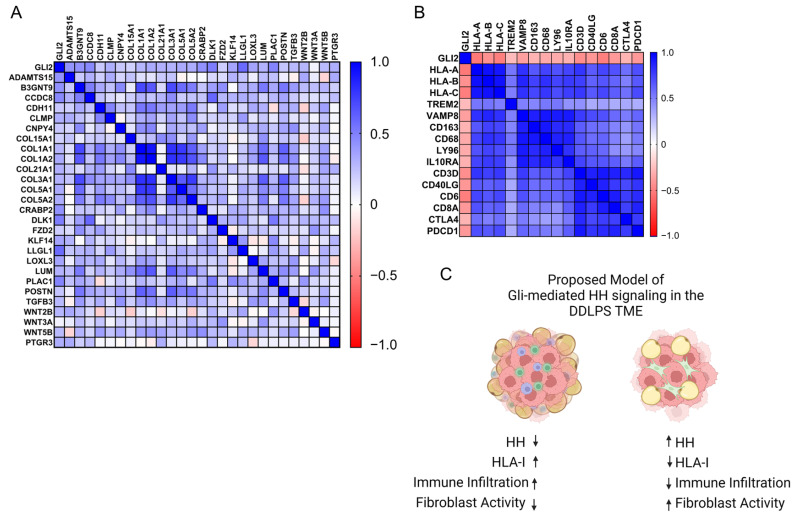
Gli2 co-expression with ECM and immune cell markers. Gene clusters associated with adipose-derived fibroblasts and immune cell populations were identified using the Human Protein Atlas. Gli2 mRNA expression was compared across these markers within the TCGA-SARC DDLPS cohort (*n* = 58) to evaluate the relationship of Gli2 between stromal cells/fibroblasts (**A**) and immune cell populations (**B**). (**C**) Proposed Model of Gli-mediated Hedgehog (HH) signaling and potential influences over the DDLPS tumor microenvironment.

Eight genes were determined to have an R of at least +0.5, and fifteen genes had an R < −0.5 and Spearman’s q-value (GSE21124 [27], TCGA-SARC [12]) or two-tailed *p*-value (GSE30929 [14]) of less than 0.05. Interestingly, a strong inverse relationship was observed between Gli2 mRNA expression and mRNA expression of HLA class I antigens (HLA-A, HLA-C) (Figure S7). Thus, we hypothesized that elevated Gli2 expression may indicate an immune-exclusive tumor environment. Using the Human Protein Atlas, we selected genes enriched in subcutaneous adipose tissue ECM remodeling, mesenchymal cells, macrophages, and T cells. Representative markers from each gene set were evaluated for correlation across the 59 TCGA-SARC DDLPS tumors. Bulk tumor RNA-seq revealed that Gli2 expression was positively correlated to markers of collagen deposition, extra cellular matrix (ECM) organization, and components of Wnt, Notch, and Transforming Growth Factor Beta signaling (Figure 6A). Additionally, we also observed a discrete inverse relationship between Gli2 expression and expression of class I HLA-antigens and markers of macrophages and T-cells within DDLPS tumors (Figure 6B). Taken together, we anticipate that Hedgehog signaling may indicate decreased HLA-I antigen presentation, resulting in lower immune cell infiltration. Additionally, higher levels of collagen expression may indicate elevated fibroblast activity and ECM deposition, further resulting in more immune exclusion from the tumor microenvironment (Figure 6C).

## 3. Discussion

In this study, Hedgehog signaling was identified as a differentially enriched pathway between adipose progenitors and mature adipocytes, as well as between well-differentiated liposarcoma (WDLPS) and dedifferentiated liposarcoma (DDLPS). Given Gli1′s genomic proximity to CDK4 and MDM2, it was anticipated that Gli1 amplification may contribute to this phenotype. Surprisingly, Gli1 amplification played less of a role than anticipated, whereas Gli2 expression appeared to be elevated in DDLPS tumors. Using co-expression analyses, Gli2 was found to have a positive relationship with cell-cycle-associated pathways and TGF-β signaling, a well-known immunosuppressive pathway, which we have previously shown can regulate Gli2 expression in the bone microenvironment [35,36,37]. Additionally, Gli2 had an inverse relationship with inflammatory signaling enrichment terms such as allograft rejection, interferon response, and JAK-STAT signaling. Upon further investigation, Gli2 expression was found to have an increasing co-expression with subcutaneous adipose tissue ECM regulators and fibroblast-associated signatures, while simultaneously having an inverse relationship with markers of adipose tissue macrophages and T cells.

These results provide novel insights into the role of Hedgehog signaling in the development of the dedifferentiated liposarcoma microenvironment, an area of research that is drastically understudied. Importantly, if Hedgehog signaling contributes to a more robust extracellular matrix in adipose tissue and adipocyte-derived tumors, then perhaps this can result in immune exclusion and a pro-tumorigenic microenvironment or fibrotic tissue formation. The most intriguing finding from our analyses is the strong inverse relationship between Gli2 expression and HLA-A and HLA-C, both class I HLA antigens. Previous work in sarcoma tumor models has shown that HLA (−) tumor cells have a greater propensity to form tumors than their HLA (+) counterparts and express a more multipotent mesenchymal transcriptional profile, often upregulating markers of bone differentiation [38]. This could also suggest an association between Gli-mediated Hedgehog signaling and tumor-initiating cell populations. Additionally, an inverse relationship between Gli2 and HLA class I antigens has been previously established in other tumor models during acquired drug resistance of tumor cells compared to wild type [39]. There is an increasing body of work linking Gli-mediated Hedgehog signaling to immune suppression [40,41,42,43,44] in the tumor microenvironment, often through cancer-associated fibroblasts; however, this has not been studied in the dedifferentiated liposarcoma tumor microenvironment. Fibroblast-specific Hedgehog signaling has been investigated in several epithelial tumor models [45,46,47,48,49] but not in sarcoma models and more specifically DDLPS. Tumor-cell-intrinsic Gli-mediated Hedgehog signaling has been identified as an oncogenic pathway in many bone-derived sarcomas [50,51,52,53,54,55,56,57], which is unsurprising, given the role of Hedgehog signaling in embryonic development and skeletal patterning. This highlights the importance of understanding the nuance of tumor biology and the biological overlap between canonical mesenchymal development and sarcoma tumor biology.

Overall, this study aimed to develop hypotheses regarding liposarcoma tumor dedifferentiation, an area that could potentially be exploited as a therapeutic vulnerability using previously collected and publicly available genomics and transcriptomics data, an underutilized resource in sarcoma research. Given the rare incidence of these tumors compared to epithelial-derived neoplasms, molecular targeted research has lagged. Here we propose that Hedgehog signaling, a driver of sarcoma formation in other tissue types [28,50,58,59,60,61], may play a role in ECM deposition and fibroblast function, while simultaneously inhibiting immune infiltration of DDLPS tumors. As a subsequent result, poor immune infiltration and therapeutic response may occur, leading us to postulate that Gli-mediated Hedgehog signaling could be an early indicator of immune exclusion and poor prognosis for DDLPS moving forward. Importantly, stromal and immune invasion have been shown to be a prognostic indicator for sarcoma tumors [62]. Our results indicate that therapeutics targeting regulatory mechanisms of cellular invasion or intrinsic tumoral stemness, a negative regulator of immune cell infiltration [63], may prove promising in the future. Based on the multidisciplinary nature of soft tissue sarcoma management, bioinformatics-based approaches may be used to supplement the extensive technological advancements that have occurred in the surgical field, allowing for clinicians to better establish protocols for post-operative monitoring. Novel clinical monitoring methods may eventually implement an integrated platform where clinical patient data can be profiled against bioinformatics platforms to better inform clinical care in the future [64].

## 4. Limitations

The authors acknowledge the limitations of data-mining-driven approaches in regard to tumor biology, particularly in the absence of terminal validation studies for the hypotheses presented above. Studies such as these are bound by the confines of the experimental methodologies performed by other laboratories, limiting the scope of conclusions that can be effectively drawn from these analyses. One example being the limited inclusion criteria for patient tumors in datasets. Variables such as this highlight the nuance of tumor heterogeneity, making it imperative that robust sample collection combined with multi-omics approaches are employed moving forward. Future work on this project will directly target Gli2 in syngeneic murine liposarcoma models to determine if Gli2-mediated Hedgehog signaling contributes to intrinsic tumor dedifferentiation and stemness or immune exclusion. In addition, single-cell RNA sequencing on DDLPS tumors should be performed to determine if Gli2 and Hedgehog signaling are expressed in tumor cells or in other mesenchymal populations, such as fibroblasts or progenitor cells, within the tumor mass. Additionally, using patient tumors to decipher the relationship between Hedgehog signaling and immune exclusion will allow for better characterization of Gli-mediated Hedgehog signaling as a potential predictor of response to immune checkpoint blockade or differentiation-based therapies.

## 5. Methods

### 5.1. Software, Databases, and Computational Tools

This work used a variety of computational tools and software to perform genomics profiling and co-expression analyses of previously collected primary sarcoma tumor tissue and normal adipose tissue. Appropriate software and web databases are listed with a brief description of its functionality in this publication in Table 1.

### 5.2. Dataset Accession and Descriptions

This study largely employed previously published and publicly available datasets to interrogate mechanisms of dedifferentiation in liposarcoma (LPS) tumors. Given the rare incidence of LPS tumors, this allowed us to expand our sample pool for genomics and transcriptomics analyses to a much larger amount than would have been achievable by our laboratory. However, this introduces caveats due to different sequencing and array platforms being used for prior publications, and as such, the authors suggest that this approach and publication be validated in greater detail prior to the use of preclinical models.

### 5.3. Genomics Profiling

Genomics analyses were performed using the CBioPortal [7,8] visualization platform. Data were uploaded previously by other laboratory groups in the publications outlined in Table 2. Samples from the TCGA-SARC [12], MSK-IMPACT [26] (Sarc_MSKCC_2022), and GSE21124 [27] (SARC_MSK_2010) datasets were filtered to only include DDLPS tumor samples. Chromosome 12q13-12q15 amplification status for these tumors was evaluated with CDK4/MDM2 and Gli1 amplification status relative to tumor-matched normal samples. Mutual exclusivity tests were performed with CBioPortal to evaluate the likelihood of amplifications co-occurring with one another.

Datasets used in this publication are shown in Table 2, while a more detailed description can be found in the supplemental information.

### 5.4. Transcriptional Profiling, Differentially Expressed Genes, and Co-Expression Analysis

Differentially expressed genes were collected using the GEO2R analytical package within the Gene Expression Omnibus (GEO). Samples were assigned based on provided descriptions from the data uploaders, and comparative statistical analyses were performed using default parameters with the significance threshold being designated at α < 0.05. Statistical comparisons for differentially expressed genes were analyzed using two-tailed *t*-test when comparing two groups, with *p*-value adjustments determined by Benjamini–Hochberg False Discovery Rate correction.

Direct mRNA expression values were collected using a variety of approaches. For datasets available through CBioPortal [7,8], genes were queried and expression values were downloaded for either normalized expression or mRNA z-score distributions across uploaded samples. For cohorts unavailable through CBioPortal [7,8], expression values were collected through uploaded matrix files accessible through GEO and evaluated using predesignated probe labels by manufacturers for the array platforms. Statistical comparisons of RNA expression values were performed using GraphPad Prism with two-tailed *t*-test or two-way ANOVA, with Tukey’s post hoc test for two groups or three groups, respectively.

Co-expression analyses were performed internally in CBioPortal [7,8] where mRNA expression values of two genes can be directly compared and the strength of the correlation can be determined using Spearman’s correlation test and false discovery correction. Co-expression analyses were performed using statistical adjustments identified by false discovery correction. This method was used to evaluate transcriptional co-expression with Gli2 within the TCGA-SARC [12] and GSE21124 [27] cohorts. In order to collect Spearman’s correlation data from GSE30929 [14], correlation tests of raw expression values were performed in GraphPad prism, due to GSE30929 [14] being unavailable in CBioPortal [7,8]. These data were used to validate the co-expression findings from the previous two cohorts, but no direct findings were extrapolated from this analysis.

### 5.5. Microarrays, Tissue RNA Sequencing, and Single-Cell RNA Sequencing

Genomics and transcriptomics profiling were performed as designated by the dataset uploaders within their respective publications. Due to variability in experimental procedures, no cross-dataset comparisons were made to eliminate confounding factors that would otherwise be introduced by experimental variability.

All microarray expression data were collected using the Affymetrix Human Genome U133A Array (GSE30929 [14], GSE21124 [27], GSE20697 [13]). Genomics array profiling was collected using the Affymetrix Mapping 250K Sty2 SNP Array (GSE21124 [27]). Sequencing was performed as outlined in original publications [12,26].

### 5.6. Gene Set Enrichment Analysis (GSEA) and Gene Ontology

GSEA was performed on expression files collected from GSE30929 [14] to determine pathway enrichment for Hedgehog signaling between WDLPS and DDLPS tumors. Files were formatted as required by GSEA software, and gene sets were collected from the molecular signatures database (MSigDB). Hallmark_Adipogenesis and Adipocytokine signaling were used as validation datasets for WDLPS tumors. KEGG_Hedgehog (Table S14), GCNP_SHH_UP_EARLY.V1_UP (Table S15), and GCNP_SHH_UP_LATE.V1_UP (Table S16) were used to evaluate enrichment of Hedgehog pathway components and downstream activity.

Gene ontology and functional enrichment analyses were performed using ShinyGO (version 0.77). ShinyGO autogenerated plots from functional enrichment or gene ontology data.

### 5.7. Statistical Analyses

Default analytical packages, statistical analyses, and parameters of previously defined software and databases were used when collecting data from CBioPortal [7,8] and GEO2R. User-performed statistical analyses were performed in GraphPad Prism (9.5.0). Statistical outliers of Gli1 mRNA expression were detected using Rout’s outlier test. Corresponding Gli2 expression values were removed from analysis. One-way ANOVA or Student’s *t*-tests were performed when comparing three or more groups or two groups, respectively. mRNA expression values were Log2-transformed, when appropriate. Spearman’s correlation analyses were performed using mRNA expression data collected from datasets and only compared internally within each dataset or patient cohort. No cross-dataset comparisons were made to eliminate confounding variables introduced by experimental procedures or analyses in other laboratories.

## Figures and Tables

**Table 1:** Software and databases used.

Software/Database	Description	Location
Gene Expression Omnibus (GEO)	Samples collected by other laboratories with results from expression or genomics profiling	https://www.ncbi.nlm.nih.gov/geo/ (Accessed on 12 August 2022)
GEO2R	Integrated comparative R script for use within GEO Can apply logarithmic transformation and post hoc analyses	https://www.ncbi.nlm.nih.gov/geo/geo2r/ (Accessed on 12 August 2022)
ShinyGO (0.77)	Functional enrichment Gene ontology analysis	http://bioinformatics.sdstate.edu/go/ (Accessed on 9 December 2022).
CBioPortal	Data visualization Genomics profiling Mutual exclusivity tests Co-expression analyses	https://www.cbioportal.org/ (Accessed on 10 June 2021)
GSEA	Gene Set Enrichment Analysis of Expression profiles uploaded by user Gene sets can be obtained from MsigDB	http://www.gsea-sigdb.org/gsea/index.jsp (Accessed on 17 April 2022)
GraphPad Prism (9.5.0)	Statistical analyses Graph generation Correlation heatmap matrices	
Biorender	Biological figure generation	https://www.biorender.com/ (Accessed on 19 February 2023)
Human Protein Atlas	Bulk and single-cell RNA sequencing for normal subcutaneous and visceral adipose tissue Gene markers for adipose tissue resident cell populations	https://www.proteinatlas.org/ENSG00000074047-GLI2/single+cell+type (Accessed on 13 June 2022)

**Table 2:** Datasets used and accession locations.

Dataset	PMID/URL	Accession Location	Description
GSE30929	21335544	Gene Expression Omnibus (GEO) GSE30929	Expression analysis by UG133A array WDLPS (*n* = 52) DDLPS (*n* = 40)
GSE20697	20887899	Gene Expression Omnibus (GEO) GSE20697	Whole-transcript expression data by UG133A array Time course of adipose stem cell differentiation
MSK-IMPACT	35705560	CBioPortal Sarcoma_MSKCC_2022	Genomics profiling by sequencing DDLPS (*n* = 167)
TCGA-SARC	29100075	CBioPortal sarc_tcga_pan_can_atlas_2018	Genomics and transcriptome profiling by sequencing DDLPS (*n* = 59)
GSE21124	20601955	CBioPortal, Sarc_mskcc (2010) Gene Expression Omnibus (GEO) GSE21122/GSE21123	Expression and genomics analysis by UG133A array DDLPS (*n* = 50) Matched Normal (*n* = 50) Normal Fat (*n* = 9)
Human Protein Atlas	25613900 28495876	https://www.proteinatlas.org/ENSG00000074047-GLI2 (Accessed on 13 June 2022)	Bulk RNA sequencing and single-cell sequencing of subcutaneous adipose tissue

## Data Availability

All data used in this work can be extrapolated from publicly available sources located in Table 2. Briefly, datasets from the Gene Expression Omnibus can be accessed at the following accession numbers: GSE30929, GSE20752, GSE21124. All CBioPortal generated data can be accessed through CBioPortal at the following studies: TCGA_SARC_2017, Sarcoma (MSK, Nat Comm. 2022), SARC (MSKCC/Broad 2010_Nat Genet.

## Supplementary Materials

The following supporting information can be downloaded at https://www.mdpi.com/article/10.3390/cancers15133360/s1, Figure S1: Hedgehog pathway components enriched in DDLPS and Adipose Progenitor Cells, Figure S2: Genomics Analysis of Gli1 amplification in two independent DDLPS patient cohorts, Figure S3: Gene Set Enrichment Analysis validation Gene Sets. GSEA was performed to evaluate hedgehog signaling enrichment between DDLPS and WDLPS tumors from the GSE30929 cohort. Adipokine signaling and adipogenesis gene sets were used for validation of WDLPS tumoral expression, Figure S4: Gli2 is enriched in mesenchymal adipose progenitors and absent from immune populations and mature adipocytes in adipose tissue, Figure S5: Gene Ontology of genes clustered with Gli2 expression during bulk tissue RNA−seq from the human protein atlas. Gli2 clustered with 335 genes, with high confidence, associated with fibroblasts across catalogued tissues, Figure S6: Gene Ontology of genes clustered with Gli2 expression during single cell RNA−sequencing form human protein atlas, Figure S7: Co−expressed Genes were evaluated across three independent DDLPS experimental cohorts. Genes with Spearman’s R > +0.5, Table S1: Gene Ontology of Genes enriched in adipose progenitors and DDLPS, Table S2: Gene Ontology of Genes enriched in adipocytes and WDLPS, Table S3: DDLPS (*n* = 40) Tumor Samples used in analyses from GSE30929 cohort, Table S4: WDLPS (*n* = 52) Tumor Samples used in analyses from GSE30929 cohort, Table S5: Time course: Human Adipose Stem Cell Differentiation, Table S6: ASC Differentiation: Full Time Course, Table S7: DDLPS Tumor Samples (*n* = 167) used in Genomics Profiling from MSK-IMPACT Cohort, Table S8: DDLPS Tumor Samples (*n* = 59) used in Genomics and Transcriptome Profiling from TCGA-SARC Cohort, Table S9: DDLPS Tumor Samples (*n* = 50) used in Genomics and Transcriptome Profiling from GSE21124, Table S10: Normal Fat Samples (*n* = 9) used in Transcriptome Profiling from GSE21124, Table S11: Matched Normal Samples (*n* = 50) used in Genomics Profiling from GSE21124, Table S12: Tumor Samples (*n* = 50) used in Genomics Profiling from GSE21124, Table S13: Genomic Alterations in Tumor Samples (*n* = 50) used in Genomics Profiling from GSE21124, Table S14: KEGG Hedgehog pathway Gene Set (hsa04340), Table S15: Gene Set GCNP_SHH_UP_EARLY.V1_UP, Table S16: Gene Set GCNP_SHH_UP_LATE.V1_UP.
