# Supplementary material for: Bioinformatics Screen Reveals Gli-Mediated Hedgehog Signaling as an Associated Pathway to Poor Immune Infiltration of Dedifferentiated Liposarcoma
Source: Cancers (Basel). 2023 Jun 27;15(13):3360. doi: 10.3390/cancers15133360 (PMC10341348; doi:10.3390/cancers15133360)

Supplemental Table S1: Gene Ontology of Genes enriched in adipose progenitors and DDLPS

Gene Sets	Enrichment FDR	nGenes	Pathway Genes	Fold Enrichment	Pathways
KEGG	1.40E−03	9	39	5.9	Various types of N−glycan biosynthesis
	1.60E−14	36	169	5.5	Protein processing in endoplasmic reticulum
	1.40E−03	10	50	5.1	N−Glycan biosynthesis
	3.20E−03	10	56	4.6	Hedgehog signaling pathway
	3.30E−02	11	93	3	TGF−beta signaling pathway
	1.40E−03	19	161	3	MicroRNAs in cancer
	5.70E−03	16	141	2.9	Ubiquitin mediated proteolysis
	3.30E−02	13	121	2.7	Thyroid hormone signaling pathway
	5.70E−03	20	202	2.5	Proteoglycans in cancer
	3.80E−02	14	143	2.5	Signaling pathways regulating pluripotency of stem cells
GO_BP	3.70E−08	24	121	5.1	Collagen fibril organization
	1.40E−09	53	450	3	Extracellular matrix organization
	1.40E−09	53	451	3	Extracellular structure organization
	1.40E−09	53	454	3	External encapsulating structure organization
	1.80E−15	125	1368	2.3	Peptidyl−amino acid modification
	4.10E−08	89	1102	2.1	Embryo development
	1.40E−09	124	1649	1.9	Plasma membrane bounded cell projection organization
	4.10E−08	109	1473	1.9	Neuron differentiation
	2.40E−09	125	1690	1.9	Cell projection organization
	4.10E−08	129	1857	1.8	Intracellular transport
Panther	3.40E−02	7	43	4.2	P00060 Ubiquitin proteasome pathway
	6.10E−03	11	75	3.8	P00059 p53 pathway
	4.20E−03	13	91	3.7	P00052 TGF−beta signaling pathway
	4.10E−02	22	286	2	P00057 Wnt signaling pathway
Reactome	1.80E−09	19	67	7.3	Collagen biosynthesis and modifying enzymes
	1.40E−09	22	90	6.3	Collagen formation
	6.20E−05	13	61	5.5	Assembly of collagen fibrils and other multimeric structures
	3.10E−05	20	133	3.8	Golgi−to−ER retrograde transport
	6.70E−06	23	154	3.8	ER to Golgi Anterograde Transport
	4.20E−11	44	305	3.7	Asparagine N−linked glycosylation
	3.80E−06	26	185	3.6	Transport to the Golgi and subsequent modification

	1.60E−05	26	203	3.3	Intra−Golgi and retrograde Golgi−to−ER traffic
	1.20E−05	41	418	2.5	Extracellular matrix organization
	3.70E−17	136	1516	2.3	Post−translational protein modification
Hallmark MSigDB	1.30E−29	55	200	7	HALLMARK EPITHELIAL MESENCHYMAL TRANSITION
	1.30E−03	9	54	4.3	HALLMARK TGF BETA SIGNALING
	3.30E−06	18	112	4.1	HALLMARK UNFOLDED PROTEIN RESPONSE
	2.20E−08	29	198	3.7	HALLMARK G2M CHECKPOINT
	7.10E−08	28	199	3.6	HALLMARK MYC TARGETS V1
	2.50E−07	27	200	3.5	HALLMARK E2F TARGETS
	1.70E−03	12	96	3.2	HALLMARK PROTEIN SECRETION
	2.60E−05	23	198	3	HALLMARK GLYCOLYSIS
	2.50E−04	21	199	2.7	HALLMARK MTORC1 SIGNALING
	1.70E−03	19	200	2.4	HALLMARK HYPOXIA

Supplemental Figure S1: Hedgehog pathway components enriched in DDLPS and Adipose Progenitor Cells. KEGG annotated Hedgehog pathway components enriched in progenitor cells and DDLPS tumors were identified and highlighted within the hedgehog signaling cascade.

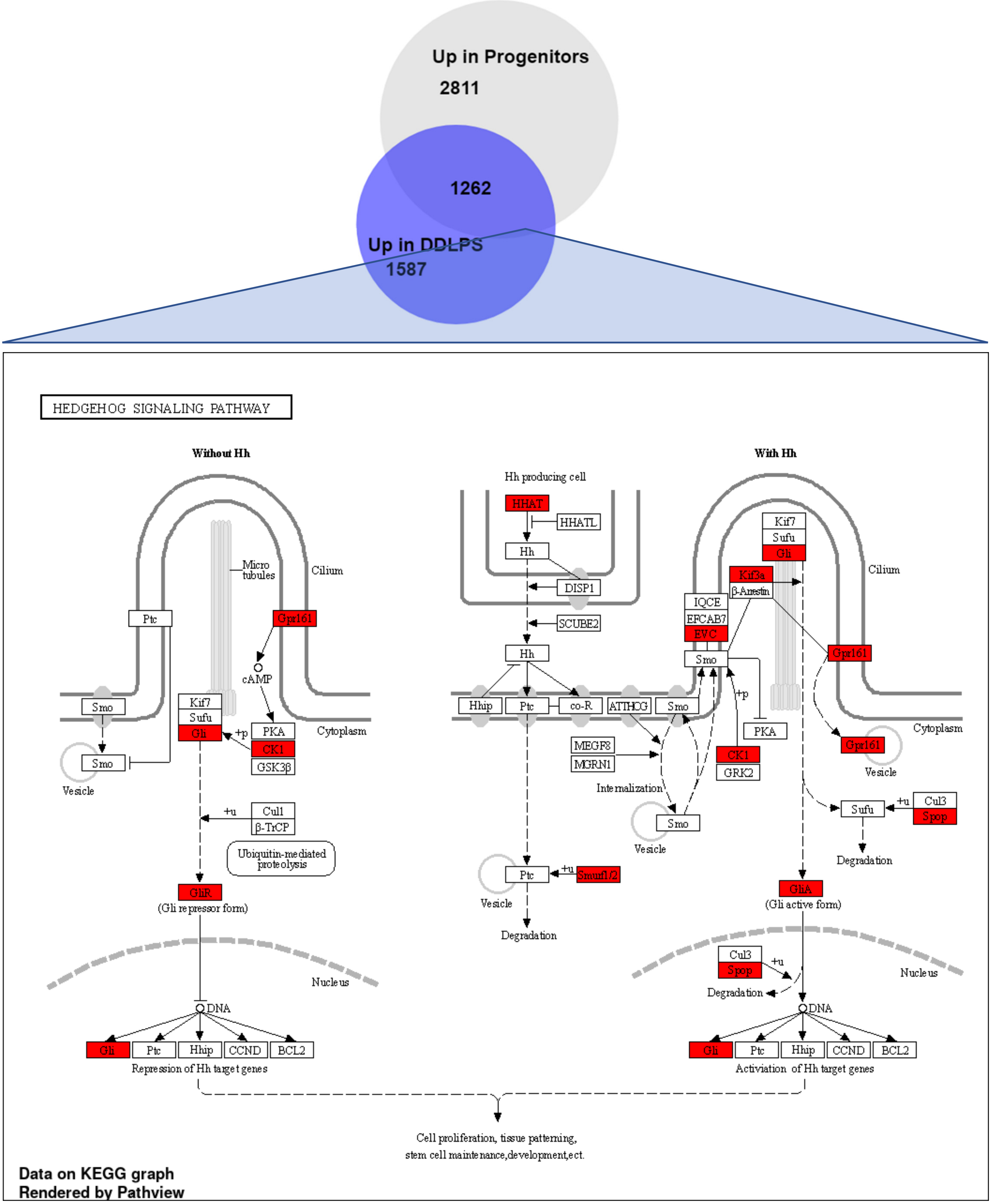

Supplemental Table S2: Gene Ontology of Genes enriched in adipocytes and WDLPS

Gene Sets	Enrichment FDR	nGenes	Pathway Genes	Fold Enrichment	Pathways
KEGG	2.90E−13	15	32	15.3	Propanoate metabolism
	8.20E−16	19	43	14.4	Fatty acid degradation
	4.20E−11	13	30	14.2	Citrate cycle (TCA cycle)
	1.10E−13	18	48	12.2	Valine, leucine and isoleucine degradation
	1.00E−11	16	47	11.1	Pyruvate metabolism
	1.70E−12	18	57	10.3	Fatty acid metabolism
	4.00E−14	22	75	9.6	PPAR signaling pathway
	3.80E−13	25	115	7.1	Carbon metabolism
	1.70E−13	26	120	7.1	AMPK signaling pathway
	1.70E−35	149	1527	3.2	Metabolic pathways
GO_BP	3.40E−25	53	266	6.5	Carboxylic acid catabolic proc.
	3.30E−34	76	414	6	Fatty acid metabolic proc.
	1.30E−43	108	675	5.2	Monocarboxylic acid metabolic proc.
	5.50E−30	83	571	4.7	Generation of precursor metabolites and energy
	7.50E−44	138	1102	4.1	Carboxylic acid metabolic proc.
	2.30E−43	139	1135	4	Oxoacid metabolic proc.
	3.30E−34	128	1178	3.5	Cellular lipid metabolic proc.
	1.40E−39	158	1555	3.3	Lipid metabolic proc.
Panther	1.50E−04	5	10	16.3	P00051 TCA cycle
	1.50E−04	5	10	16.3	P02772 Pyruvate metabolism
	1.70E−05	7	16	14.3	P04372 5−Hydroxytryptamine degradation
	1.10E−02	3	8	12.2	P02762 Pentose phosphate pathway
	1.10E−03	7	35	6.5	P00033 Insulin/IGF pathway−protein kinase B signaling cascade
	1.10E−03	8	47	5.6	P00048 PI3 kinase pathway
	2.00E−04	14	114	4	P00018 EGF receptor signaling pathway
	1.10E−02	10	103	3.2	P00021 FGF signaling pathway
	1.50E−03	15	161	3	P00034 Integrin signaling pathway
	2.20E−03	15	169	2.9	P06959 CCKR signaling map
Reactome	8.30E−07	7	11	20.8	Mitochondrial fatty acid beta−oxidation of saturated fatty acids
	3.40E−10	14	37	12.4	Mitochondrial Fatty Acid Beta−Oxidation
	1.30E−07	11	31	11.6	Pyruvate metabolism
	9.90E−11	17	55	10.1	Pyruvate metabolism and Citric Acid TCA cycle
	4.80E−08	13	43	9.9	Signaling by Retinoic Acid
	3.50E−21	41	183	7.3	Fatty acid metabolism
	8.90E−14	34	197	5.6	Metabolism of vitamins and cofactors
	1.30E−07	20	123	5.3	Metabolism of water−soluble vitamins and cofactors

	2.60E−10	28	178	5.1	The citric acid TCA cycle and respiratory electron transport
	2.10E−27	94	783	3.9	Metabolism of lipids
Hallmark MSigDB	5.20E−75	86	199	14.1	HALLMARK ADIPOGENESIS
	4.30E−33	48	158	9.9	HALLMARK FATTY ACID METABOLISM
	4.90E−26	46	200	7.5	HALLMARK OXIDATIVE PHOSPHORYLATION
	1.40E−10	21	112	6.1	HALLMARK BILE ACID METABOLISM
	1.20E−17	37	200	6	HALLMARK XENOBIOTIC METABOLISM
	1.70E−09	19	104	6	HALLMARK PEROXISOME
	1.60E−13	32	200	5.2	HALLMARK MYOGENESIS
	8.50E−13	31	200	5.1	HALLMARK ESTROGEN RESPONSE LATE
	4.40E−12	30	200	4.9	HALLMARK HYPOXIA
	2.20E−11	29	200	4.7	HALLMARK ESTROGEN RESPONSE EARLY

Supplemental Figure S2: Genomics Analysis of Gli1 amplification in two independent DDLPS patient cohorts. (A) Amplification status of CDK4, MDM2, Gli1 with subsequent mutual exclusivity tests in TCGA−SARC DDLPS tumors and (B) GSE21124 DDLPS tumors. (C) Gene expression of Gli1 relative to CDK4 and MDM2 genomic status in DDLPS tumors from (C) TCGA−SARC cohort and (D) GSE21124 cohort. (n.s.: not significant, *p<0.05)

A

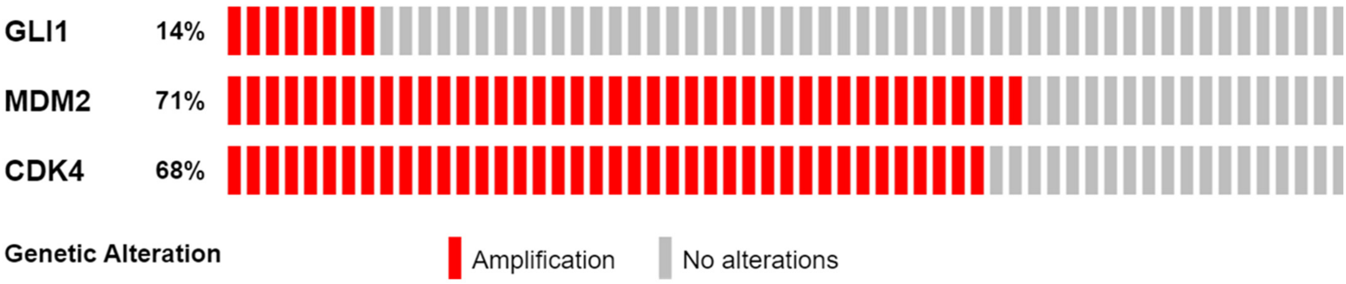

A	B	Neither	A Not B	B Not A	Both	Log2 Odds Ratio	p-Value	q-Value	Tendency
**MDM2**	**CDK4**	**17**	**2**	**0**	**40**	**>3**	**<0.001**	**<0.001**	**Co-occurrence**
GLI1	CDK4	19	0	32	8	>3	0.035	0.052	Co-occurrence
GLI1	MDM2	17	0	34	8	>3	0.053	0.053	Co-occurrence

B

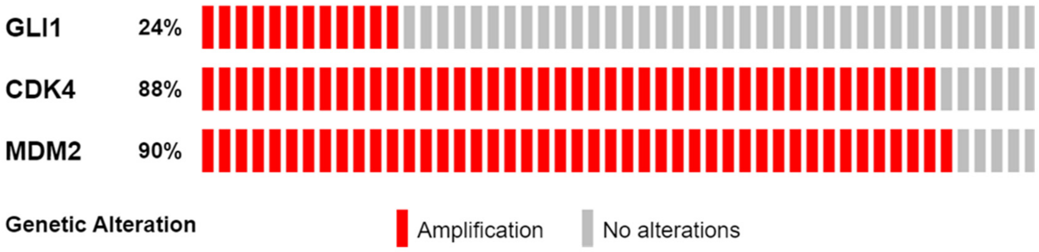

A	B	Neither	A Not B	B Not A	Both	Log2 Odds Ratio	p-Value	q-Value	Tendency
**CDK4**	**MDM2**	**5**	**0**	**1**	**44**	**>3**	**<0.001**	**<0.001**	**Co-occurrence**

Gli1 not profiled

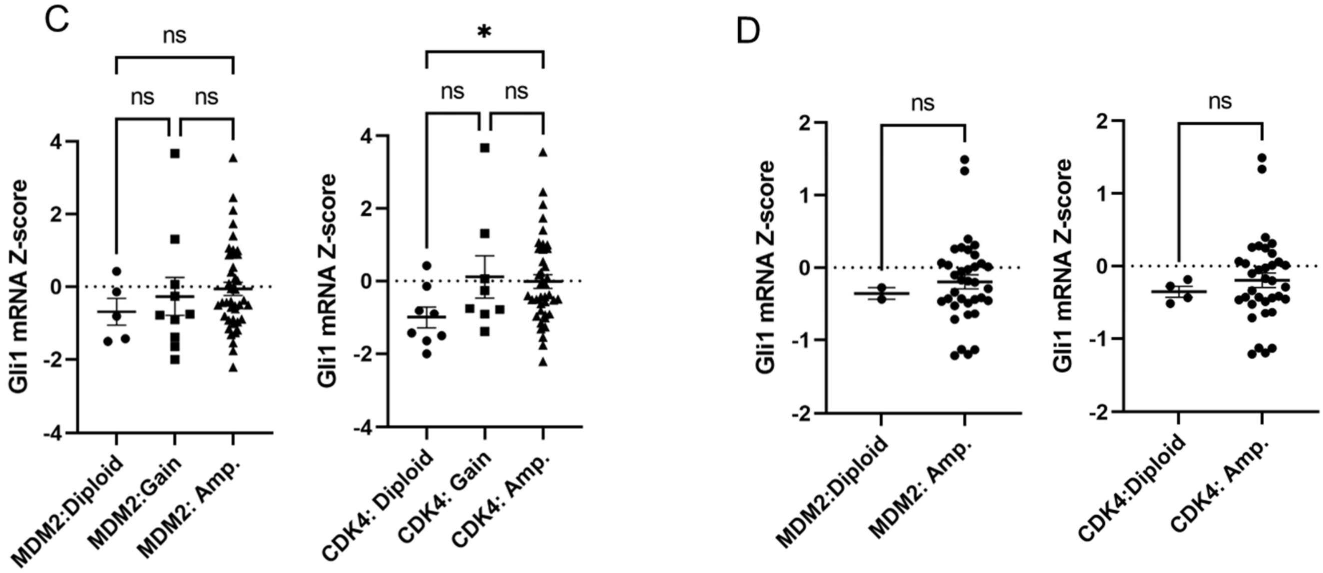

Supplemental Figure S3: Gene Set Enrichment Analysis validation Gene Sets**.** GSEA was performed to evaluate hedgehog signaling enrichment between DDLPS and WDLPS tumors from the GSE30929 cohort. Adipokine signaling and adipogenesis gene sets were used for validation of WDLPS tumoral expression.

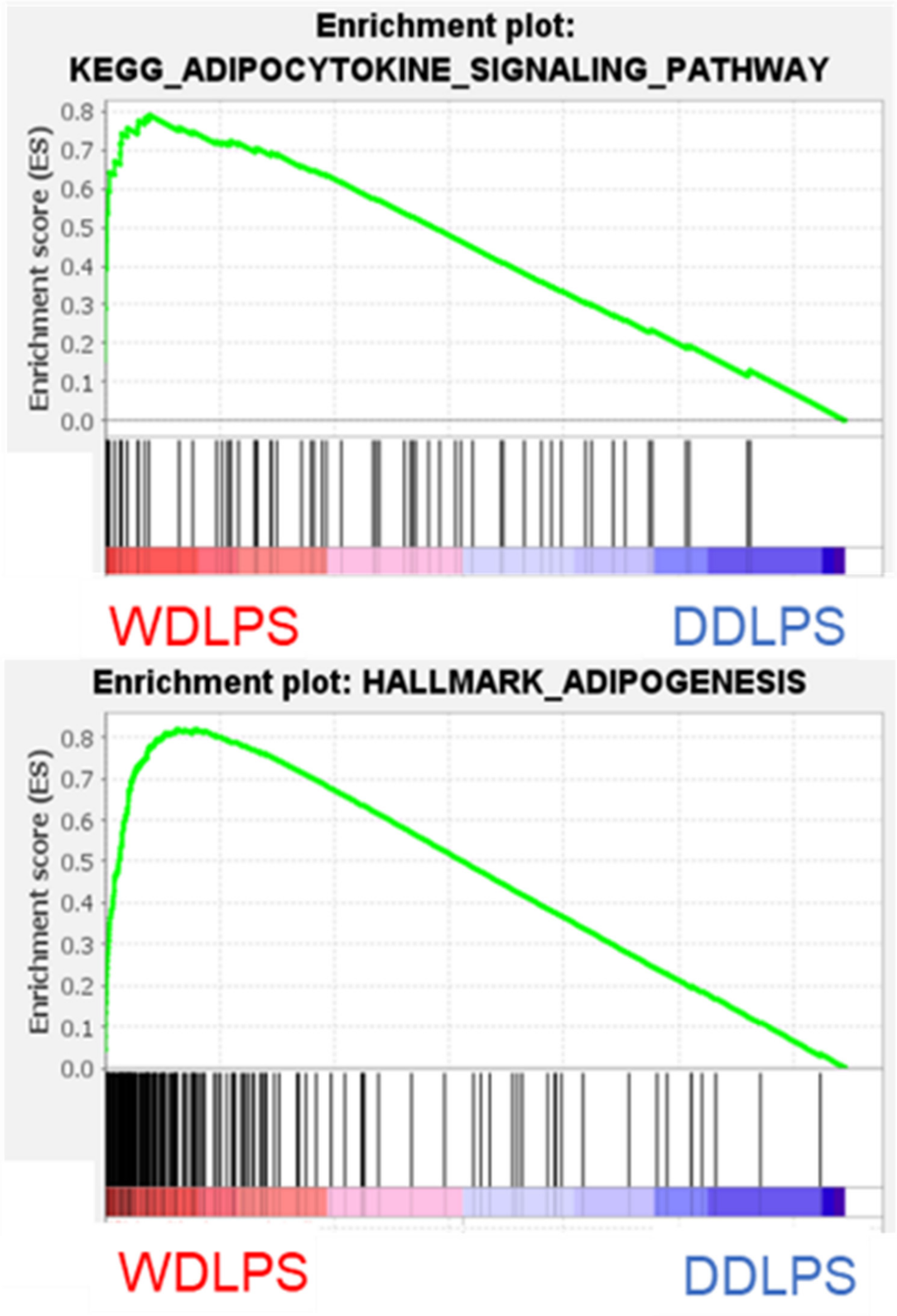

Supplemental Figure S4: Gli2 is enriched in mesenchymal adipose progenitors and absent from immune populations and mature adipocytes in adipose tissue. Gli2 expression data in normal subcutaneous adipose tissues were evaluated using the human protein atlas (A). Gli2 expression decreased during adipocyte differentiation (GSE20752) (B). Whole tissue sequencing co−expression analysis experimental outline (C). Quantification of Gli2 enrichment score based on cell marker expression in subcutaneous adipose tissue (D). Single cell RNA sequencing distribution of Gli2 expression (E). Cell population cluster expression of Gli2 shown in E (F). (n.s.: not significant**p<0.01, ***p<0.001)

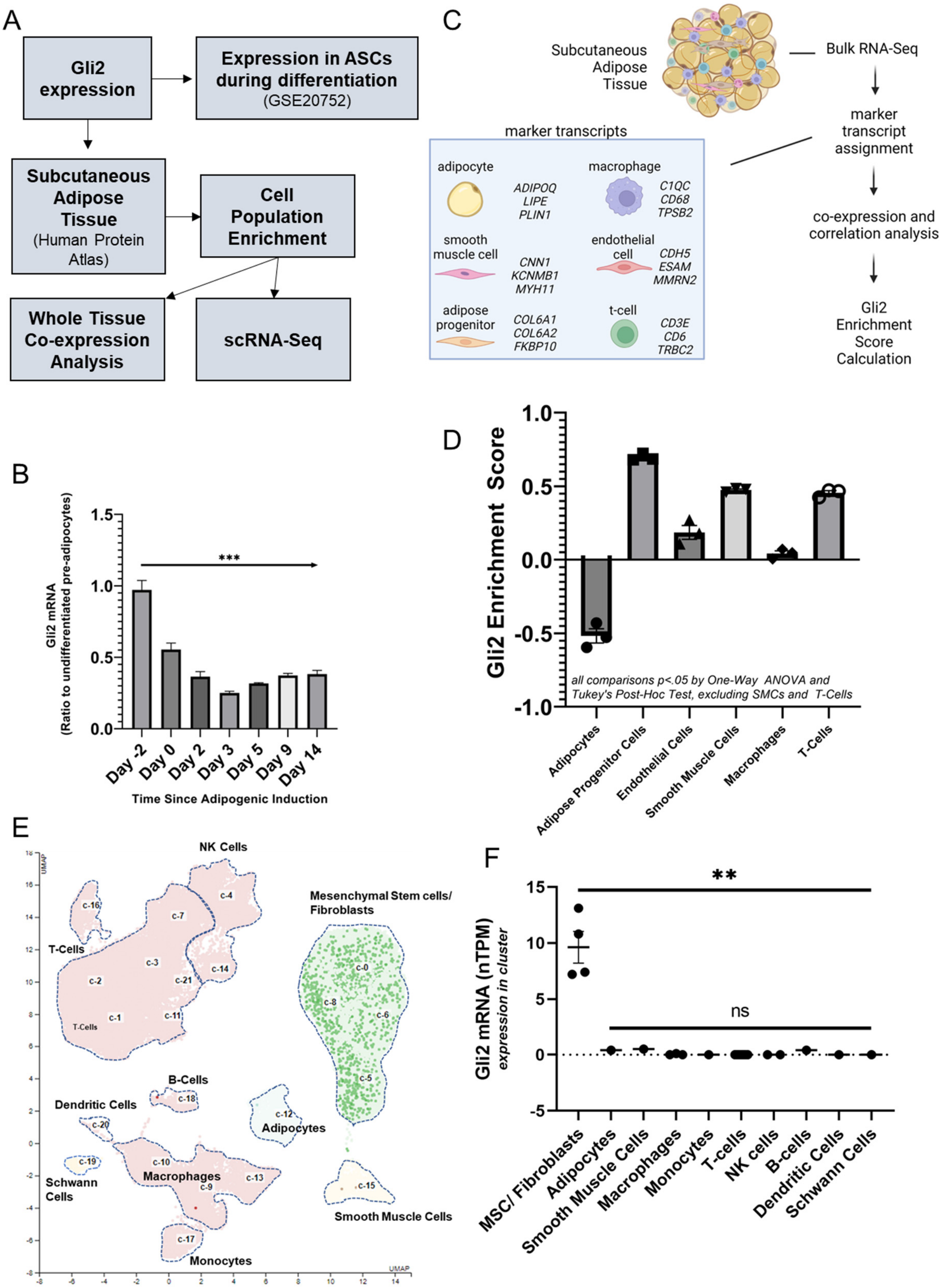

Supplemental Figure S5: Gene Ontology of genes clustered with Gli2 expression during bulk tissue RNA−seq from the human protein atlas. Gli2 clustered with 335 genes, with high confidence, associated with fibroblasts across catalogued tissues. These genes were associated with transcriptional regulation, embryonic development, mesenchymal differentiation, and stem cell population maintenance. (MF: molecular function, CC: cellular component, BP: biological process)

Clustering Methods: https://www.proteinatlas.org/about/assays+annotation#gene_clustering_rna

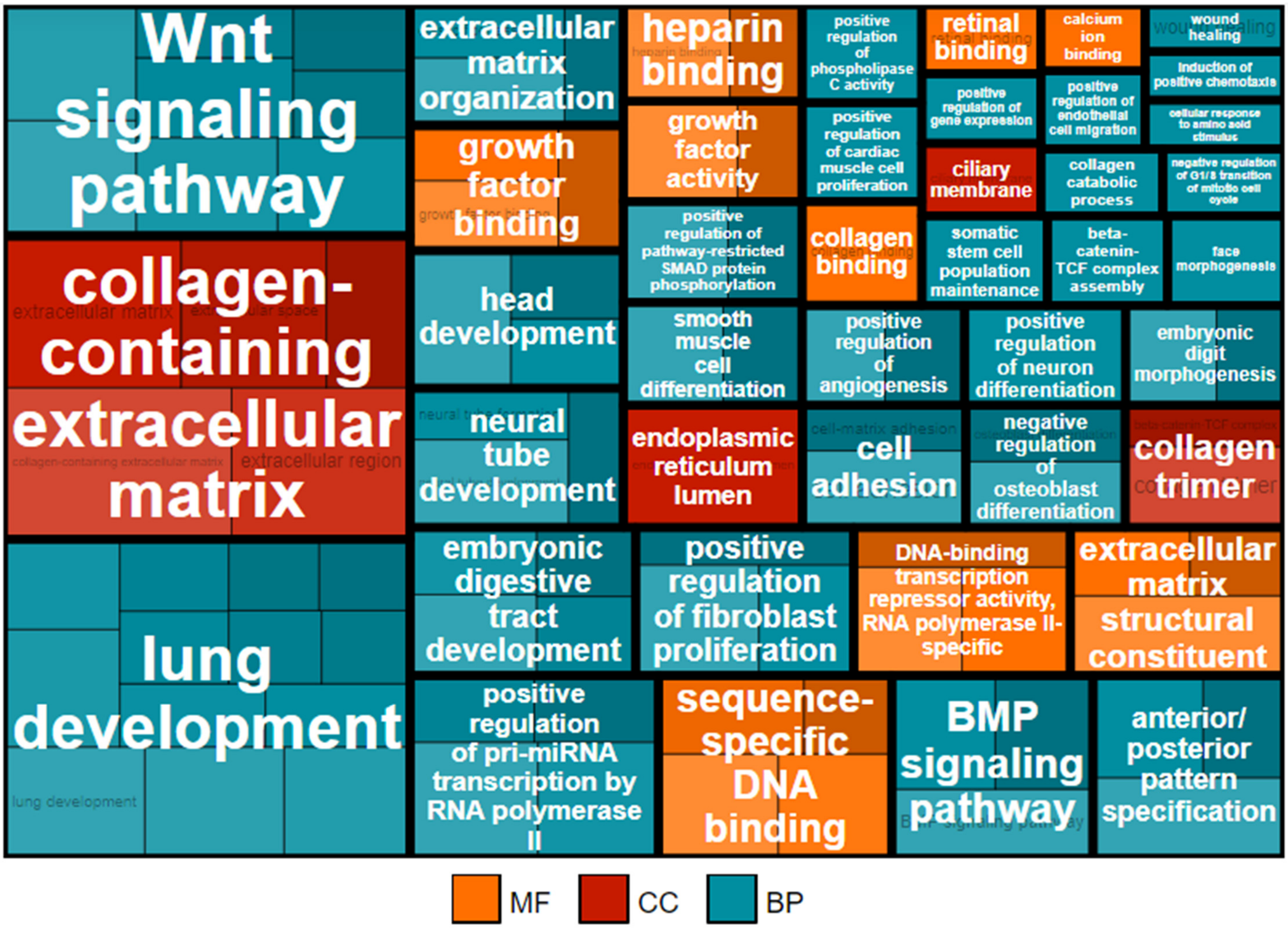

Supplemental Figure S6: Gene Ontology of genes clustered with Gli2 expression during single cell RNA−sequencing form human protein atlas. Gli2 clustered with 187 genes within Connective Tissue Cells, specifically ECM organization. (MF: molecular function, CC: cellular component, BP: biological process). Genes were functionally associated with mesenchymal differentiation, ECM development and organization, wound healing, and morphogenesis.

Clustering Methods: https://www.proteinatlas.org/about/assays+annotation#gene_clustering_rna

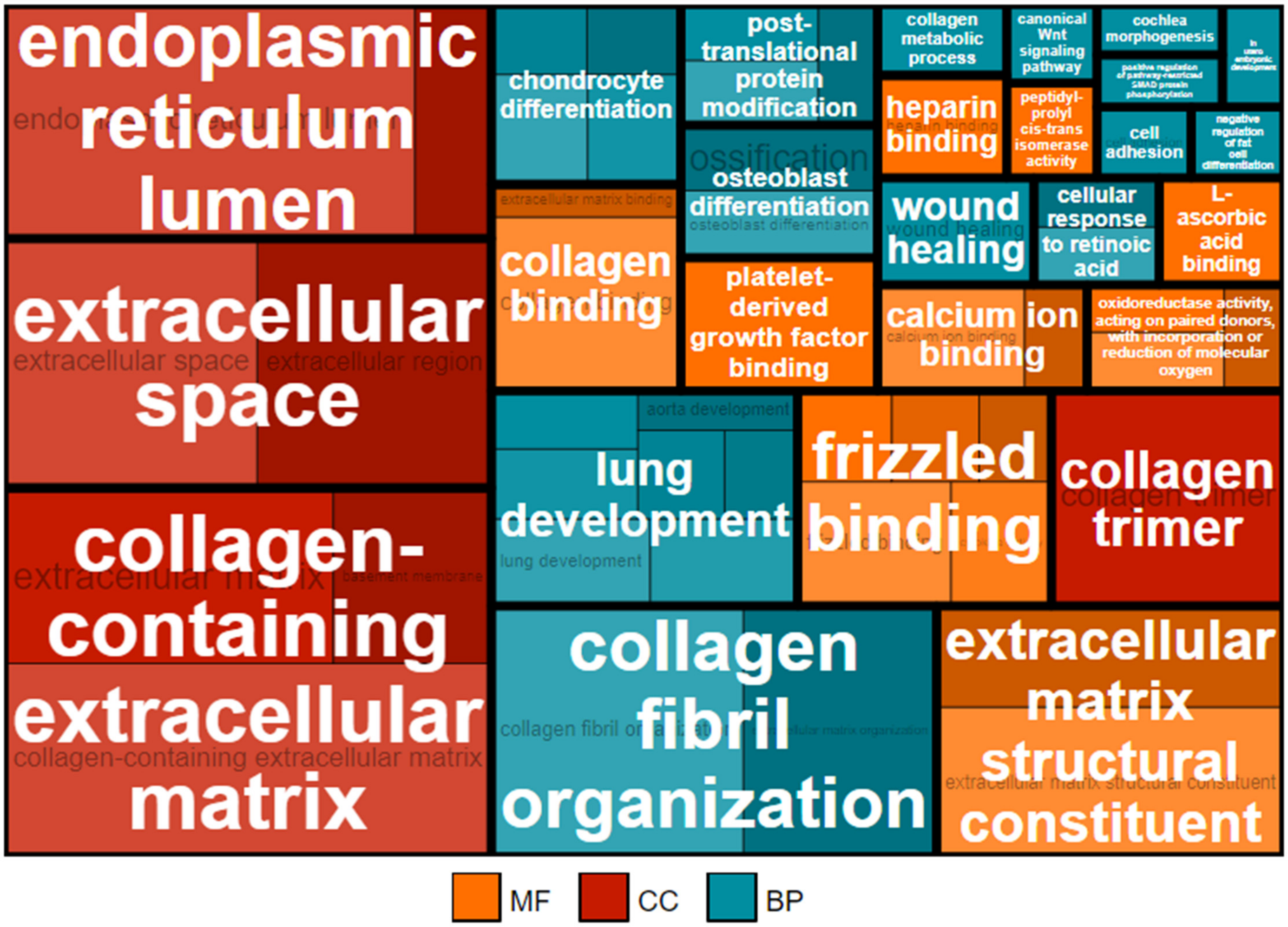

Supplemental Figure S7: Co−expressed Genes were evaluated across three independent DDLPS experimental cohorts**.** Genes with Spearman's R > +0.5 (A). Genes with Spearman's R <−0.5 (B). Heatmap plot of correlation distribution across 3 cohorts of overlapping correlated genes (C). (MSKCC_2012: GSE21124)

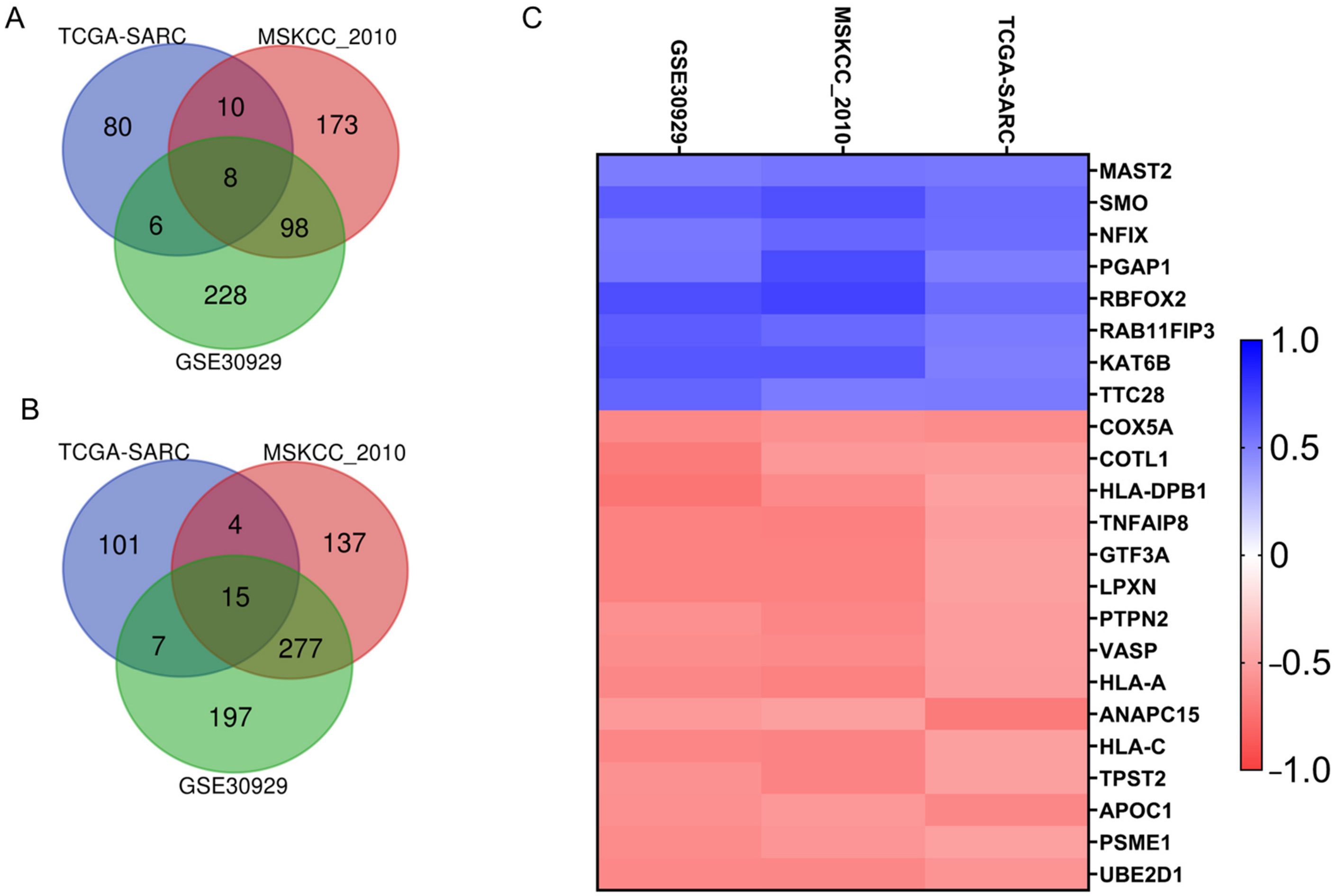

Supplement: Supplementary file 1 [file cancers-15-03360-s001.zip › Supplemental Data final.pdf]
