# Supplementary material for: Bioinformatics Screen Reveals Gli-Mediated Hedgehog Signaling as an Associated Pathway to Poor Immune Infiltration of Dedifferentiated Liposarcoma
Source: Cancers (Basel). 2023 Jun 27;15(13):3360. doi: 10.3390/cancers15133360 (PMC10341348; doi:10.3390/cancers15133360)
Supplement: Supplementary file 1 [file cancers-15-03360-s001.zip › Supplemental tables_final.pdf]

Table S3: DDLPS (n=40) Tumor Samples used in analyses from GSE30929 cohort

Whole-Transcript Expression Data for Liposarcoma (HG-U133A array)

Gobble RM, Qin LX, Brill ER, Angeles CV et al. Expression profiling of liposarcoma yields a multigene predictor of patient outcome and identifies genes that contribute to liposarcomagenesis. Cancer Res 2011 Apr 1;71(7):2697-705. PMID: 21335544

Accession URL: https://www.ncbi.nlm.nih.gov/geo/query/acc.cgi?acc=GSE30929

GEO Sample	Sample Identifier	GEO Sample	Sample Identifier
GSM766533	Sarcoma tumor DD0728	GSM766553	Sarcoma tumor DD610
GSM766534	Sarcoma tumor DD103	GSM766554	Sarcoma tumor DD611
GSM766535	Sarcoma tumor DD112	GSM766555	Sarcoma tumor DD6213
GSM766536	Sarcoma tumor DD172	GSM766556	Sarcoma tumor DD639
GSM766537	Sarcoma tumor DD176	GSM766557	Sarcoma tumor DD668
GSM766538	Sarcoma tumor DD188	GSM766558	Sarcoma tumor DD670
GSM766539	Sarcoma tumor DD216	GSM766559	Sarcoma tumor DD672
GSM766540	Sarcoma tumor DD226	GSM766560	Sarcoma tumor DD69
GSM766541	Sarcoma tumor DD330	GSM766561	Sarcoma tumor DD712
GSM766542	Sarcoma tumor DD344	GSM766562	Sarcoma tumor DD714
GSM766543	Sarcoma tumor DD382	GSM766563	Sarcoma tumor DD715
GSM766544	Sarcoma tumor DD391	GSM766564	Sarcoma tumor DD718
GSM766545	Sarcoma tumor DD442	GSM766565	Sarcoma tumor DD719
GSM766546	Sarcoma tumor DD454	GSM766566	Sarcoma tumor DD7533
GSM766547	Sarcoma tumor DD456	GSM766567	Sarcoma tumor DD8379
GSM766548	Sarcoma tumor DD474	GSM766568	Sarcoma tumor DD88
GSM766549	Sarcoma tumor DD506	GSM766569	Sarcoma tumor WD439*
GSM766550	Sarcoma tumor DD513	GSM766570	Sarcoma tumor PL243*
GSM766551	Sarcoma tumor DD595	GSM766571	Sarcoma tumor WD432*
GSM766552	Sarcoma tumor DD597	GSM766572	Sarcoma tumor DD3516

*Labeled as DDLPS in GEO by authors **

Table S4: WDLPS (n=52) Tumor Samples used in analyses from GSE30929 cohort

Whole-Transcript Expression Data for Liposarcoma (HG-U133A array)

Gobble RM, Qin LX, Brill ER, Angeles CV et al. Expression profiling of liposarcoma yields a multigene predictor of patient outcome and identifies genes that contribute to liposarcomagenesis. Cancer Res 2011 Apr 1;71(7):2697-705. PMID: 21335544

Accession URL: https://www.ncbi.nlm.nih.gov/geo/query/acc.cgi?acc=GSE30929

GEO Sample	Sample Identifier	GEO Sample	Sample Identifier
GSM766621	Sarcoma tumor WD401	GSM766647	Sarcoma tumor WD450
GSM766622	Sarcoma tumor WD602	GSM766648	Sarcoma tumor WD459
GSM766623	Sarcoma tumor WD72	GSM766649	Sarcoma tumor WD463
GSM766624	Sarcoma tumor ALT476	GSM766650	Sarcoma tumor WD468
GSM766625	Sarcoma tumor ALT479	GSM766651	Sarcoma tumor WD472
GSM766626	Sarcoma tumor ALT486	GSM766652	Sarcoma tumor WD499
GSM766627	Sarcoma tumor ALT497	GSM766653	Sarcoma tumor WD4994
GSM766628	Sarcoma tumor ALT500	GSM766654	Sarcoma tumor WD510
GSM766629	Sarcoma tumor WD444	GSM766655	Sarcoma tumor WD558
GSM766630	Sarcoma tumor WD700	GSM766656	Sarcoma tumor WD5700
GSM766631	Sarcoma tumor WD702	GSM766657	Sarcoma tumor WD589
GSM766632	Sarcoma tumor WD0611	GSM766658	Sarcoma tumor WD60
GSM766633	Sarcoma tumor WD194	GSM766659	Sarcoma tumor WD6233
GSM766634	Sarcoma tumor WD199	GSM766660	Sarcoma tumor WD687
GSM766635	Sarcoma tumor WD200	GSM766661	Sarcoma tumor WD688
GSM766636	Sarcoma tumor WD221	GSM766662	Sarcoma tumor WD691
GSM766637	Sarcoma tumor WD286	GSM766663	Sarcoma tumor WD696
GSM766638	Sarcoma tumor WD356	GSM766664	Sarcoma tumor WD701
GSM766639	Sarcoma tumor WD359	GSM766665	Sarcoma tumor WD705
GSM766640	Sarcoma tumor WD368	GSM766666	Sarcoma tumor WD706
GSM766641	Sarcoma tumor WD375	GSM766667	Sarcoma tumor WD721
GSM766642	Sarcoma tumor WD380	GSM766668	Sarcoma tumor WD724
GSM766643	Sarcoma tumor WD410	GSM766669	Sarcoma tumor WD725
GSM766644	Sarcoma tumor WD418	GSM766670	Sarcoma tumor WD9343
GSM766645	Sarcoma tumor WD419	GSM766671	Sarcoma tumor WD9370
GSM766646	Sarcoma tumor WD426	GSM766672	Sarcoma tumor WD0429

Table S5: Time course: Human Adipose Stem Cell Differentiation

Whole-Transcript Expression Data (HG-U133A array)

Mikkelsen TS, Xu Z, Zhang X, Wang L et al. Comparative epigenomic analysis of murine and human adipogenesis. Cell 2010 Oct 1;143(1):156-69. PMID: 20887899

Accession URL: https://www.ncbi.nlm.nih.gov/geo/query/acc.cgi?acc=GSE20752 (Subseries GSE20697)

**Undifferentiated Adipose Stem Cells**		**Differentiated Adipocytes**	
**GEO Sample**	**Sample Identifier**	**GEO Sample**	**Sample Identifier**
GSM519587	hASC_t1_noFGF_rep1	GSM519601	hASC_t8_rep1
GSM519588	hASC_t1_noFGF_rep2	GSM519602	hASC_t8_rep2
GSM519589	hASC_t1_rep1	GSM519603	hASC_t9_rep1
GSM519590	hASC_t1_rep2	GSM519604	hASC_t9_rep2

Table S6.

ASC Differentiation: Full Time Course

GSM519590	hASC_t1_rep2
GSM519591	hASC_t2_rep1
GSM519592	hASC_t2_rep2
GSM519593	hASC_t3_rep1
GSM519594	hASC_t4_rep1
GSM519595	hASC_t4_rep2
GSM519596	hASC_t5_rep1
GSM519597	hASC_t5_rep2
GSM519598	hASC_t6_rep1
GSM519599	hASC_t6_rep2
GSM519600	hASC_t7_rep1
GSM519601	hASC_t8_rep1
GSM519602	hASC_t8_rep2
GSM519603	hASC_t9_rep1
GSM519604	hASC_t9_rep2

Table S7: DDLPS Tumor Samples (n=167) used in Genomics Profiling from MSK-IMPACT Cohort

Genomics Profiling of Sarcoma Tumors by Sequencing

Nacev BA, Sanchez-Vega F, Smith SA, Antonescu CR, Rosenbaum E, Shi H, Tang C, Socci ND, Rana S, Gularte-Mérida R, Zehir A, Gounder MM, Bowler TG, Luthra A, Jadeja B, Okada A, Strong JA, Stoller J, Chan JE, Chi P, D'Angelo SP, Dickson MA, Kelly CM, Keohan ML, Movva S, Thornton K, Meyers PA, Wexler LH, Slotkin EK, Glade Bender JL, Shukla NN, Hensley ML, Healey JH, La Quaglia MP, Alektiar KM, Crago AM, Yoon SS, Untch BR, Chiang S, Agaram NP, Hameed MR, Berger MF, Solit DB, Schultz N, Ladanyi M, Singer S, Tap WD. Clinical sequencing of soft tissue and bone sarcomas delineates diverse genomic landscapes and potential therapeutic targets. Nat Commun. 2022 Jun 15;13(1):3405. doi: 10.1038/s41467-022-30453-x. PMID: 35705560; PMCID: PMC9200818.

Accession URL: https://www.cbioportal.org/study/summary?id=sarcoma_mskcc_2022

Queried Genes: CDK4, MDM2, GLI1

Study ID	Sample ID	Patient ID	MDM2	CDK4	GLI1
sarcoma_mskcc_2022	P-0000146-T01-IM3	P-0000146	AMP (driver)	AMP (driver)	not profiled
sarcoma_mskcc_2022	P-0000796-T01-IM3	P-0000796	AMP (driver)	AMP (driver)	not profiled
sarcoma_mskcc_2022	P-0001056-T01-IM3	P-0001056	AMP (driver)	AMP (driver)	not profiled
sarcoma_mskcc_2022	P-0001596-T01-IM3	P-0001596	AMP (driver)	AMP (driver)	not profiled
sarcoma_mskcc_2022	P-0001612-T01-IM3	P-0001612	AMP (driver)	AMP (driver)	not profiled
sarcoma_mskcc_2022	P-0001870-T01-IM3	P-0001870	AMP (driver)	AMP (driver)	not profiled
sarcoma_mskcc_2022	P-0002301-T01-IM3	P-0002301	AMP (driver)	AMP (driver)	not profiled
sarcoma_mskcc_2022	P-0002326-T02-IM5	P-0002326	AMP (driver)	AMP (driver)	no alteration
sarcoma_mskcc_2022	P-0002393-T01-IM3	P-0002393	AMP (driver)	AMP (driver)	not profiled
sarcoma_mskcc_2022	P-0002919-T01-IM3	P-0002919	AMP (driver)	AMP (driver)	not profiled
sarcoma_mskcc_2022	P-0003144-T01-IM5	P-0003144	AMP (driver)	AMP (driver)	no alteration
sarcoma_mskcc_2022	P-0003171-T01-IM5	P-0003171	AMP (driver)	AMP (driver)	no alteration
sarcoma_mskcc_2022	P-0003228-T01-IM5	P-0003228	AMP (driver)	AMP (driver)	AMP (driver)
sarcoma_mskcc_2022	P-0003688-T01-IM5	P-0003688	AMP (driver)	AMP (driver)	no alteration
sarcoma_mskcc_2022	P-0003874-T01-IM5	P-0003874	AMP (driver)	AMP (driver)	no alteration
sarcoma_mskcc_2022	P-0004657-T02-IM5	P-0004657	AMP (driver)	AMP (driver)	no alteration
sarcoma_mskcc_2022	P-0004682-T01-IM5	P-0004682	AMP (driver)	AMP (driver)	no alteration
sarcoma_mskcc_2022	P-0004819-T01-IM5	P-0004819	AMP (driver)	AMP (driver)	no alteration
sarcoma_mskcc_2022	P-0005175-T01-IM5	P-0005175	AMP (driver)	AMP (driver)	no alteration
sarcoma_mskcc_2022	P-0005323-T01-IM5	P-0005323	AMP (driver)	AMP (driver)	no alteration
sarcoma_mskcc_2022	P-0005396-T01-IM5	P-0005396	AMP (driver)	AMP (driver)	no alteration
sarcoma_mskcc_2022	P-0005478-T01-IM5	P-0005478	AMP (driver)	AMP (driver)	no alteration
sarcoma_mskcc_2022	P-0006258-T01-IM5	P-0006258	AMP (driver)	AMP (driver)	AMP (driver)
sarcoma_mskcc_2022	P-0006284-T01-IM5	P-0006284	AMP (driver)	AMP (driver)	no alteration
sarcoma_mskcc_2022	P-0006373-T01-IM5	P-0006373	AMP (driver)	AMP (driver)	no alteration
sarcoma_mskcc_2022	P-0006577-T01-IM5	P-0006577	AMP (driver)	AMP (driver)	no alteration
sarcoma_mskcc_2022	P-0006594-T01-IM5	P-0006594	AMP (driver)	AMP (driver)	Q508*, AMP (driver)
sarcoma_mskcc_2022	P-0006605-T01-IM5	P-0006605	AMP (driver)	no alteration	no alteration
sarcoma_mskcc_2022	P-0006799-T01-IM5	P-0006799	AMP (driver)	AMP (driver)	AMP (driver)
sarcoma_mskcc_2022	P-0006982-T01-IM5	P-0006982	AMP (driver)	AMP (driver)	no alteration
sarcoma_mskcc_2022	P-0007111-T01-IM5	P-0007111	AMP (driver)	AMP (driver)	no alteration
sarcoma_mskcc_2022	P-0007536-T01-IM5	P-0007536	AMP (driver)	AMP (driver)	AMP (driver)
sarcoma_mskcc_2022	P-0007735-T01-IM5	P-0007735	AMP (driver)	no alteration	no alteration

sarcoma_mskcc_2022	P-0008708-T01-IM5	P-0008708	AMP (driver)	AMP (driver)	no alteration
sarcoma_mskcc_2022	P-0008866-T01-IM5	P-0008866	AMP (driver)	AMP (driver)	no alteration
sarcoma_mskcc_2022	P-0009147-T01-IM5	P-0009147	no alteration	AMP (driver)	AMP (driver)
sarcoma_mskcc_2022	P-0009246-T01-IM5	P-0009246	AMP (driver)	AMP (driver)	GLI1-ATXN7L3B Fusion
sarcoma_mskcc_2022	P-0009471-T01-IM5	P-0009471	AMP (driver)	AMP (driver)	AMP (driver)
sarcoma_mskcc_2022	P-0009653-T01-IM5	P-0009653	AMP (driver)	AMP (driver)	AMP (driver)
sarcoma_mskcc_2022	P-0009968-T01-IM5	P-0009968	AMP (driver)	AMP (driver)	no alteration
sarcoma_mskcc_2022	P-0009987-T01-IM5	P-0009987	AMP (driver)	no alteration	no alteration
sarcoma_mskcc_2022	P-0010314-T01-IM5	P-0010314	AMP (driver)	AMP (driver)	no alteration
sarcoma_mskcc_2022	P-0010468-T04-IM6	P-0010468	AMP (driver)	AMP (driver)	no alteration
sarcoma_mskcc_2022	P-0010589-T01-IM5	P-0010589	AMP (driver)	AMP (driver)	AMP (driver)
sarcoma_mskcc_2022	P-0011094-T01-IM5	P-0011094	AMP (driver)	AMP (driver)	no alteration
sarcoma_mskcc_2022	P-0011123-T01-IM5	P-0011123	AMP (driver)	AMP (driver)	no alteration
sarcoma_mskcc_2022	P-0013205-T01-IM5	P-0013205	AMP (driver)	AMP (driver)	no alteration
sarcoma_mskcc_2022	P-0013316-T01-IM5	P-0013316	AMP (driver)	AMP (driver)	no alteration
sarcoma_mskcc_2022	P-0013365-T01-IM5	P-0013365	AMP (driver)	AMP (driver)	no alteration
sarcoma_mskcc_2022	P-0013948-T01-IM5	P-0013948	AMP (driver)	AMP (driver)	no alteration
sarcoma_mskcc_2022	P-0013959-T01-IM5	P-0013959	AMP (driver)	AMP (driver)	no alteration
sarcoma_mskcc_2022	P-0014231-T01-IM6	P-0014231	AMP (driver)	AMP (driver)	AMP (driver)
sarcoma_mskcc_2022	P-0014876-T01-IM6	P-0014876	AMP (driver)	AMP (driver)	no alteration
sarcoma_mskcc_2022	P-0015631-T01-IM6	P-0015631	AMP (driver)	AMP (driver)	no alteration
sarcoma_mskcc_2022	P-0015682-T01-IM6	P-0015682	AMP (driver)	AMP (driver)	no alteration
sarcoma_mskcc_2022	P-0016132-T01-IM6	P-0016132	AMP (driver)	AMP (driver)	no alteration
sarcoma_mskcc_2022	P-0016178-T02-IM6	P-0016178	AMP (driver)	AMP (driver)	AMP (driver)
sarcoma_mskcc_2022	P-0017035-T01-IM6	P-0017035	AMP (driver)	AMP (driver)	no alteration
sarcoma_mskcc_2022	P-0017423-T01-IM6	P-0017423	AMP (driver)	AMP (driver)	no alteration
sarcoma_mskcc_2022	P-0017718-T01-IM6	P-0017718	AMP (driver)	AMP (driver)	AMP (driver)
sarcoma_mskcc_2022	P-0018754-T01-IM6	P-0018754	AMP (driver)	AMP (driver)	AMP (driver)
sarcoma_mskcc_2022	P-0018868-T01-IM6	P-0018868	AMP (driver)	AMP (driver)	no alteration
sarcoma_mskcc_2022	P-0019498-T01-IM6	P-0019498	AMP (driver)	AMP (driver)	AMP (driver)
sarcoma_mskcc_2022	P-0019508-T01-IM6	P-0019508	AMP (driver)	AMP (driver)	no alteration
sarcoma_mskcc_2022	P-0019922-T01-IM6	P-0019922	AMP (driver)	AMP (driver)	GLI1-LGR5 Fusion
sarcoma_mskcc_2022	P-0020491-T01-IM6	P-0020491	AMP (driver)	AMP (driver)	no alteration
sarcoma_mskcc_2022	P-0020909-T01-IM6	P-0020909	AMP (driver)	AMP (driver)	no alteration
sarcoma_mskcc_2022	P-0021032-T01-IM6	P-0021032	AMP (driver)	AMP (driver)	AMP (driver)
sarcoma_mskcc_2022	P-0021313-T01-IM6	P-0021313	AMP (driver)	AMP (driver)	no alteration
sarcoma_mskcc_2022	P-0021618-T01-IM6	P-0021618	AMP (driver)	AMP (driver)	AMP (driver)
sarcoma_mskcc_2022	P-0021640-T01-IM6	P-0021640	AMP (driver)	no alteration	no alteration
sarcoma_mskcc_2022	P-0021838-T01-IM6	P-0021838	AMP (driver)	AMP (driver)	no alteration
sarcoma_mskcc_2022	P-0022148-T01-IM6	P-0022148	AMP (driver)	AMP (driver)	no alteration
sarcoma_mskcc_2022	P-0022486-T01-IM6	P-0022486	AMP (driver)	AMP (driver), CDK4-MIR5700 Fusion	AMP (driver)
sarcoma_mskcc_2022	P-0022591-T01-IM6	P-0022591	AMP (driver)	AMP (driver)	no alteration
sarcoma_mskcc_2022	P-0022861-T02-IM6	P-0022861	AMP (driver)	AMP (driver)	AMP (driver)

sarcoma_mskcc_2022	P-0022968-T01-IM6	P-0022968	AMP (driver)	AMP (driver)	no alteration
sarcoma_mskcc_2022	P-0023059-T01-IM6	P-0023059	AMP (driver)	AMP (driver)	AMP (driver)
sarcoma_mskcc_2022	P-0024246-T01-IM6	P-0024246	AMP (driver)	AMP (driver)	no alteration
sarcoma_mskcc_2022	P-0025900-T01-IM6	P-0025900	AMP (driver)	AMP (driver)	AMP (driver)
sarcoma_mskcc_2022	P-0026061-T01-IM6	P-0026061	AMP (driver)	AMP (driver)	AMP (driver)
sarcoma_mskcc_2022	P-0026368-T01-IM6	P-0026368	AMP (driver)	AMP (driver)	no alteration
sarcoma_mskcc_2022	P-0026423-T01-IM6	P-0026423	AMP (driver)	AMP (driver)	no alteration
sarcoma_mskcc_2022	P-0026492-T01-IM6	P-0026492	AMP (driver)	AMP (driver)	no alteration
sarcoma_mskcc_2022	P-0026859-T01-IM6	P-0026859	AMP (driver)	AMP (driver)	AMP (driver)
sarcoma_mskcc_2022	P-0026862-T01-IM6	P-0026862	D179E, AMP (driver)	AMP (driver)	no alteration
sarcoma_mskcc_2022	P-0026913-T01-IM6	P-0026913	AMP (driver)	AMP (driver)	AMP (driver)
sarcoma_mskcc_2022	P-0027938-T01-IM6	P-0027938	AMP (driver)	AMP (driver)	no alteration
sarcoma_mskcc_2022	P-0028063-T01-IM6	P-0028063	AMP (driver)	AMP (driver)	no alteration
sarcoma_mskcc_2022	P-0028166-T01-IM6	P-0028166	AMP (driver)	AMP (driver)	no alteration
sarcoma_mskcc_2022	P-0028347-T01-IM6	P-0028347	AMP (driver)	AMP (driver)	no alteration
sarcoma_mskcc_2022	P-0028519-T01-IM6	P-0028519	AMP (driver)	AMP (driver)	AMP (driver)
sarcoma_mskcc_2022	P-0028595-T02-IM6	P-0028595	AMP (driver)	AMP (driver)	no alteration
sarcoma_mskcc_2022	P-0028636-T01-IM6	P-0028636	AMP (driver)	AMP (driver)	AMP (driver)
sarcoma_mskcc_2022	P-0028678-T01-IM6	P-0028678	AMP (driver)	AMP (driver)	no alteration
sarcoma_mskcc_2022	P-0028915-T01-IM6	P-0028915	AMP (driver)	AMP (driver)	no alteration
sarcoma_mskcc_2022	P-0028991-T01-IM6	P-0028991	AMP (driver)	AMP (driver)	no alteration
sarcoma_mskcc_2022	P-0029373-T01-IM6	P-0029373	AMP (driver)	AMP (driver)	AMP (driver)
sarcoma_mskcc_2022	P-0030087-T01-IM6	P-0030087	AMP (driver)	AMP (driver)	no alteration
sarcoma_mskcc_2022	P-0030122-T01-IM6	P-0030122	AMP (driver)	AMP (driver)	no alteration
sarcoma_mskcc_2022	P-0030137-T02-IM6	P-0030137	AMP (driver)	AMP (driver)	no alteration
sarcoma_mskcc_2022	P-0030245-T01-IM6	P-0030245	AMP (driver)	AMP (driver)	no alteration
sarcoma_mskcc_2022	P-0030267-T01-IM6	P-0030267	AMP (driver)	AMP (driver)	AMP (driver)
sarcoma_mskcc_2022	P-0030333-T01-IM6	P-0030333	AMP (driver)	AMP (driver)	no alteration
sarcoma_mskcc_2022	P-0030514-T01-IM6	P-0030514	AMP (driver)	AMP (driver)	no alteration
sarcoma_mskcc_2022	P-0030720-T01-IM6	P-0030720	AMP (driver)	AMP (driver)	no alteration
sarcoma_mskcc_2022	P-0030722-T01-IM6	P-0030722	AMP (driver)	AMP (driver)	no alteration
sarcoma_mskcc_2022	P-0030733-T01-IM6	P-0030733	AMP (driver)	AMP (driver)	AMP (driver)
sarcoma_mskcc_2022	P-0030885-T01-IM6	P-0030885	AMP (driver)	AMP (driver)	no alteration
sarcoma_mskcc_2022	P-0031044-T01-IM6	P-0031044	AMP (driver)	AMP (driver)	AMP (driver)
sarcoma_mskcc_2022	P-0031152-T01-IM6	P-0031152	AMP (driver)	AMP (driver)	no alteration
sarcoma_mskcc_2022	P-0031396-T01-IM6	P-0031396	AMP (driver)	AMP (driver)	no alteration
sarcoma_mskcc_2022	P-0031452-T01-IM6	P-0031452	AMP (driver)	AMP (driver)	no alteration
sarcoma_mskcc_2022	P-0031514-T01-IM6	P-0031514	AMP (driver)	AMP (driver)	no alteration
sarcoma_mskcc_2022	P-0032007-T01-IM6	P-0032007	AMP (driver)	AMP (driver)	AMP (driver)
sarcoma_mskcc_2022	P-0032043-T01-IM6	P-0032043	AMP (driver)	AMP (driver)	no alteration
sarcoma_mskcc_2022	P-0032096-T01-IM6	P-0032096	AMP (driver)	AMP (driver)	AMP (driver)
sarcoma_mskcc_2022	P-0032211-T02-IM6	P-0032211	AMP (driver), Protein Fusion: mid-exon {TPH2:MDM2}	AMP (driver)	AMP (driver)
sarcoma_mskcc_2022	P-0032459-T01-IM6	P-0032459	AMP (driver)	AMP (driver)	AMP (driver)

sarcoma_mskcc_2022	P-0033008-T01-IM6	P-0033008	AMP (driver)	AMP (driver)	no alteration
sarcoma_mskcc_2022	P-0033724-T01-IM6	P-0033724	AMP (driver)	AMP (driver)	no alteration
sarcoma_mskcc_2022	P-0033898-T01-IM6	P-0033898	AMP (driver)	AMP (driver)	no alteration
sarcoma_mskcc_2022	P-0033953-T01-IM6	P-0033953	AMP (driver)	AMP (driver)	no alteration
sarcoma_mskcc_2022	P-0033963-T01-IM6	P-0033963	AMP (driver)	AMP (driver)	no alteration
sarcoma_mskcc_2022	P-0034080-T02-IM6	P-0034080	AMP (driver)	AMP (driver)	AMP (driver)
sarcoma_mskcc_2022	P-0034262-T01-IM6	P-0034262	AMP (driver)	no alteration	no alteration
sarcoma_mskcc_2022	P-0034379-T01-IM6	P-0034379	AMP (driver)	AMP (driver)	no alteration
sarcoma_mskcc_2022	P-0034631-T01-IM6	P-0034631	AMP (driver)	AMP (driver)	AMP (driver)
sarcoma_mskcc_2022	P-0034684-T01-IM6	P-0034684	AMP (driver)	AMP (driver)	no alteration
sarcoma_mskcc_2022	P-0035694-T01-IM6	P-0035694	AMP (driver)	AMP (driver)	no alteration
sarcoma_mskcc_2022	P-0036132-T01-IM6	P-0036132	AMP (driver)	AMP (driver)	no alteration
sarcoma_mskcc_2022	P-0036282-T01-IM6	P-0036282	AMP (driver)	AMP (driver)	no alteration
sarcoma_mskcc_2022	P-0036526-T01-IM6	P-0036526	AMP (driver)	AMP (driver)	AMP (driver)
sarcoma_mskcc_2022	P-0036558-T01-IM6	P-0036558	AMP (driver)	AMP (driver)	no alteration
sarcoma_mskcc_2022	P-0036662-T01-IM6	P-0036662	AMP (driver)	AMP (driver)	no alteration
sarcoma_mskcc_2022	P-0036698-T01-IM6	P-0036698	AMP (driver)	AMP (driver)	no alteration
sarcoma_mskcc_2022	P-0037337-T01-IM6	P-0037337	AMP (driver)	AMP (driver)	AMP (driver)
sarcoma_mskcc_2022	P-0037728-T01-IM6	P-0037728	AMP (driver)	AMP (driver)	no alteration
sarcoma_mskcc_2022	P-0038298-T01-IM6	P-0038298	AMP (driver)	AMP (driver)	no alteration
sarcoma_mskcc_2022	P-0038773-T01-IM6	P-0038773	AMP (driver)	AMP (driver)	no alteration
sarcoma_mskcc_2022	P-0039139-T01-IM6	P-0039139	AMP (driver)	AMP (driver)	no alteration
sarcoma_mskcc_2022	P-0039480-T01-IM6	P-0039480	AMP (driver)	AMP (driver)	AMP (driver)
sarcoma_mskcc_2022	P-0040091-T01-IM6	P-0040091	AMP (driver)	AMP (driver)	no alteration
sarcoma_mskcc_2022	P-0040815-T01-IM6	P-0040815	AMP (driver)	AMP (driver), Protein Fusion: mid-exon {CDK4:DCC}	no alteration
sarcoma_mskcc_2022	P-0041422-T01-IM6	P-0041422	AMP (driver)	AMP (driver)	no alteration
sarcoma_mskcc_2022	P-0041513-T01-IM6	P-0041513	AMP (driver), Protein Fusion: mid-exon {ABCC4:MDM2}	AMP (driver)	no alteration
sarcoma_mskcc_2022	P-0041627-T01-IM6	P-0041627	AMP (driver)	AMP (driver)	no alteration
sarcoma_mskcc_2022	P-0042355-T01-IM6	P-0042355	AMP (driver)	AMP (driver)	no alteration
sarcoma_mskcc_2022	P-0042527-T01-IM6	P-0042527	AMP (driver)	AMP (driver)	no alteration
sarcoma_mskcc_2022	P-0042879-T01-IM6	P-0042879	E346K, E421K, K475N, AMP (driver)	AMP (driver)	no alteration
sarcoma_mskcc_2022	P-0043065-T01-IM6	P-0043065	AMP (driver)	AMP (driver)	no alteration
sarcoma_mskcc_2022	P-0043148-T01-IM6	P-0043148	AMP (driver)	AMP (driver)	no alteration
sarcoma_mskcc_2022	P-0043632-T01-IM6	P-0043632	AMP (driver)	AMP (driver)	AMP (driver), Protein Fusion: mid-exon {GLI1:ZFAND3}
sarcoma_mskcc_2022	P-0043703-T01-IM6	P-0043703	AMP (driver)	AMP (driver)	AMP (driver)
sarcoma_mskcc_2022	P-0044140-T01-IM6	P-0044140	AMP (driver)	AMP (driver)	no alteration
sarcoma_mskcc_2022	P-0044575-T01-IM6	P-0044575	AMP (driver)	AMP (driver)	no alteration
sarcoma_mskcc_2022	P-0044607-T01-IM6	P-0044607	AMP (driver)	AMP (driver)	no alteration

sarcoma_mskcc_2022	P-0044741-T01-IM6	P-0044741	AMP (driver)	AMP (driver)	no alteration
sarcoma_mskcc_2022	P-0044885-T01-IM6	P-0044885	AMP (driver)	AMP (driver)	no alteration
sarcoma_mskcc_2022	P-0044911-T01-IM6	P-0044911	AMP (driver)	AMP (driver)	no alteration
sarcoma_mskcc_2022	P-0045656-T01-IM6	P-0045656	AMP (driver)	AMP (driver)	no alteration
sarcoma_mskcc_2022	P-0045729-T01-IM6	P-0045729	AMP (driver)	AMP (driver)	no alteration
sarcoma_mskcc_2022	P-0045832-T01-IM6	P-0045832	AMP (driver), Protein Fusion: out of frame {MDM2:GNPTAB}	AMP (driver)	no alteration
sarcoma_mskcc_2022	P-0024168-T01-IM6	P-0024168	no alteration	no alteration	no alteration
sarcoma_mskcc_2022	P-0026209-T01-IM6	P-0026209	no alteration	no alteration	no alteration
sarcoma_mskcc_2022	P-0027663-T01-IM6	P-0027663	no alteration	no alteration	no alteration
sarcoma_mskcc_2022	P-0030777-T01-IM6	P-0030777	no alteration	no alteration	no alteration

Table S8: DDLPS Tumor Samples (n=59) used in Genomics and Transcriptome Profiling from TCGA-SARC Cohort

Profiling of Sarcoma Tumors by Sequencing

Accession URL: https://www.cbioportal.org/study/summary?id=sarc_tcga_pan_can_atlas_2018

Queried Genes: MDM2, CDK4, GLI1, GLI2

Study ID	Patient ID	MDM2	CDK4	GLI1	GLI2
sarc_tcga_pan_can_atlas_2018	TCGA-3B-A9HJ	AMP (driver)	AMP (driver), FRS2-CDK4 fusion	no alteration	no alteration
sarc_tcga_pan_can_atlas_2018	TCGA-3B-A9HL	AMP (driver)	AMP (driver)	no alteration	no alteration
sarc_tcga_pan_can_atlas_2018	TCGA-3B-A9HO	AMP (driver)	AMP (driver), CDK4-PICSAR fusion, RILPL1-CDK4 fusion	no alteration	no alteration
sarc_tcga_pan_can_atlas_2018	TCGA-3R-A8YX	AMP (driver)	AMP (driver)	no alteration	no alteration
sarc_tcga_pan_can_atlas_2018	TCGA-DX-A1KU	AMP (driver), MDM2-DDR2 fusion	AMP (driver)	AMP (driver)	no alteration
sarc_tcga_pan_can_atlas_2018	TCGA-DX-A1KW	AMP (driver)	AMP (driver)	no alteration	no alteration
sarc_tcga_pan_can_atlas_2018	TCGA-DX-A1KX	no alteration	no alteration	no alteration	V796M
sarc_tcga_pan_can_atlas_2018	TCGA-DX-A1KZ	AMP (driver)	AMP (driver)	no alteration	no alteration
sarc_tcga_pan_can_atlas_2018	TCGA-DX-A1L0	AMP (driver)	AMP (driver)	no alteration	no alteration
sarc_tcga_pan_can_atlas_2018	TCGA-DX-A1L1	AMP (driver)	no alteration	no alteration	no alteration
sarc_tcga_pan_can_atlas_2018	TCGA-DX-A1L3	AMP (driver), MDM2-FMO6P fusion	AMP (driver)	no alteration	no alteration
sarc_tcga_pan_can_atlas_2018	TCGA-DX-A1L4	AMP (driver)	AMP (driver)	no alteration	no alteration
sarc_tcga_pan_can_atlas_2018	TCGA-DX-A23R	AMP (driver)	AMP (driver)	AMP (driver)	no alteration
sarc_tcga_pan_can_atlas_2018	TCGA-DX-A23T	AMP (driver)	AMP (driver)	no alteration	no alteration
sarc_tcga_pan_can_atlas_2018	TCGA-DX-A23V	AMP (driver)	AMP (driver), DAB1-CDK4 fusion, OMA1-CDK4 fusion	no alteration	no alteration
sarc_tcga_pan_can_atlas_2018	TCGA-DX-A23Y	AMP (driver), MDM2-C12orf50 fusion, NT5E-MDM2 fusion	AMP (driver)	no alteration	no alteration
sarc_tcga_pan_can_atlas_2018	TCGA-DX-A23Z	AMP (driver)	AMP (driver)	HOMDEL	no alteration
sarc_tcga_pan_can_atlas_2018	TCGA-DX-A240	MDM2-SLAMF9 fusion	no alteration	no alteration	no alteration
sarc_tcga_pan_can_atlas_2018	TCGA-DX-A2J0	AMP (driver)	AMP (driver)	no alteration	no alteration

sarc_tcga_pan_can_atlas_2018	TCGA-DX-A2J1	AMP (driver), MDM2-NAV3 fusion	AMP (driver)	no alteration	no alteration
sarc_tcga_pan_can_atlas_2018	TCGA-DX-A2J4	AMP (driver)	AMP (driver)	no alteration	no alteration
sarc_tcga_pan_can_atlas_2018	TCGA-DX-A3LS	L230F, AMP (driver)	AMP (driver)	no alteration	no alteration
sarc_tcga_pan_can_atlas_2018	TCGA-DX-A3LT	AMP (driver)	AMP (driver)	no alteration	no alteration
sarc_tcga_pan_can_atlas_2018	TCGA-DX-A3LU	AMP (driver)	AMP (driver)	no alteration	no alteration
sarc_tcga_pan_can_atlas_2018	TCGA-DX-A3LW	AMP (driver)	AMP (driver)	no alteration	no alteration
sarc_tcga_pan_can_atlas_2018	TCGA-DX-A3LY	AMP (driver)	AMP (driver)	no alteration	no alteration
sarc_tcga_pan_can_atlas_2018	TCGA-DX-A3M1	S221C, AMP (driver), MDM2-SYK fusion	AMP (driver)	no alteration	no alteration
sarc_tcga_pan_can_atlas_2018	TCGA-DX-A3U5	AMP (driver)	AMP (driver)	no alteration	no alteration
sarc_tcga_pan_can_atlas_2018	TCGA-DX-A3U6	AMP (driver)	AMP (driver)	AMP (driver)	no alteration
sarc_tcga_pan_can_atlas_2018	TCGA-DX-A48N	MDM2-POLR3B fusion	no alteration	no alteration	no alteration
sarc_tcga_pan_can_atlas_2018	TCGA-DX-A6BE	AMP (driver)	AMP (driver)	AMP (driver)	no alteration
sarc_tcga_pan_can_atlas_2018	TCGA-DX-A6BF	AMP (driver)	AMP (driver)	no alteration	no alteration
sarc_tcga_pan_can_atlas_2018	TCGA-DX-A6BH	AMP (driver)	AMP (driver)	no alteration	no alteration
sarc_tcga_pan_can_atlas_2018	TCGA-DX-A7EI	AMP (driver)	AMP (driver)	no alteration	no alteration
sarc_tcga_pan_can_atlas_2018	TCGA-DX-A7ES	AMP (driver)	AMP (driver)	no alteration	no alteration
sarc_tcga_pan_can_atlas_2018	TCGA-DX-AB2F	AMP (driver), MDM2-FRS2 fusion	AMP (driver)	AMP (driver)	no alteration
sarc_tcga_pan_can_atlas_2018	TCGA-DX-AB2G	AMP (driver), MDM2-DIP2B fusion	AMP (driver)	no alteration	no alteration
sarc_tcga_pan_can_atlas_2018	TCGA-DX-AB2H	AMP (driver)	AMP (driver), ZFC3H1-CDK4 fusion	AMP (driver)	no alteration
sarc_tcga_pan_can_atlas_2018	TCGA-DX-AB35	AMP (driver)	AMP (driver)	no alteration	no alteration
sarc_tcga_pan_can_atlas_2018	TCGA-DX-AB36	AMP (driver)	AMP (driver), CDK4-ANO5 fusion	no alteration	no alteration
sarc_tcga_pan_can_atlas_2018	TCGA-DX-AB37	AMP (driver)	no alteration	no alteration	no alteration
sarc_tcga_pan_can_atlas_2018	TCGA-DX-AB3A	AMP (driver), MDM2-FGF18 fusion	AMP (driver)	no alteration	no alteration
sarc_tcga_pan_can_atlas_2018	TCGA-FX-A2QS	AMP (driver)	AMP (driver)	AMP (driver)	no alteration

sarc_tcga_pan_can_atlas_2018	TCGA-FX-A3NK	MDM2-MYRFL fusion	no alteration	R496L	no alteration
sarc_tcga_pan_can_atlas_2018	TCGA-IF-A3RQ	MDM2-ZCCHC5 fusion	no alteration	no alteration	no alteration
sarc_tcga_pan_can_atlas_2018	TCGA-SG-A6Z7	AMP (driver), LINC-PINT-MDM2 fusion	AMP (driver)	AMP (driver)	no alteration
sarc_tcga_pan_can_atlas_2018	TCGA-WK-A8XO	AMP (driver), MDM2-IL22 fusion, MDM2-PTPRR fusion	AMP (driver)	no alteration	no alteration
sarc_tcga_pan_can_atlas_2018	TCGA-3B-A9HI	no alteration	no alteration	no alteration	no alteration
sarc_tcga_pan_can_atlas_2018	TCGA-DX-A1KY	no alteration	no alteration	no alteration	no alteration
sarc_tcga_pan_can_atlas_2018	TCGA-DX-A1L2	no alteration	no alteration	no alteration	no alteration
sarc_tcga_pan_can_atlas_2018	TCGA-DX-A23U	no alteration	no alteration	no alteration	no alteration
sarc_tcga_pan_can_atlas_2018	TCGA-DX-A2IZ	no alteration	no alteration	no alteration	no alteration
sarc_tcga_pan_can_atlas_2018	TCGA-DX-A3M2	no alteration	no alteration	no alteration	no alteration
sarc_tcga_pan_can_atlas_2018	TCGA-DX-A6BG	no alteration	no alteration	no alteration	no alteration
sarc_tcga_pan_can_atlas_2018	TCGA-DX-A6BK	no alteration	no alteration	no alteration	no alteration
sarc_tcga_pan_can_atlas_2018	TCGA-MO-A47P	no alteration	no alteration	no alteration	no alteration
sarc_tcga_pan_can_atlas_2018	TCGA-QQ-A5VA	no alteration	no alteration	no alteration	no alteration
sarc_tcga_pan_can_atlas_2018	TCGA-WK-A8XQ	no alteration	no alteration	no alteration	no alteration
sarc_tcga_pan_can_atlas_2018	TCGA-Z4-A9VC	no alteration	no alteration	no alteration	no alteration

Table S9: DDLPS Tumor Samples (n=50) used in Genomics and Transcriptome Profiling from GSE21124

Profiling of Sarcoma Tumors by Array

Barretina J, Taylor BS, Banerji S, Ramos AH et al. Subtype-specific genomic alterations define new targets for soft-tissue sarcoma therapy. *Nat Genet* 2010 Aug;42(8):715-21. PMID: 20601955

Accession URL(s): https://www.cbioportal.org/study/summary?id=sarc_mskcc
https://www.ncbi.nlm.nih.gov/geo/query/acc.cgi?acc=GSE21124

Queried Genes: MDM2, CDK4, GLI1, GLI2

Expression Profiling (GSE21122, CBioPortal)

GEO Sample	Sample Identifier	GEO Sample	Sample Identifier
GSM528276	Sarcoma tumor PT1DD	GSM528303	Sarcoma tumor PT31DD
GSM528277	Sarcoma tumor PT2DD	GSM528304	Sarcoma tumor PT32DD
GSM528278	Sarcoma PT3DD	GSM528305	Sarcoma tumor PT33DD
GSM528282	Sarcoma tumor PT7DD	GSM528306	Sarcoma tumor PT34DD
GSM528283	Sarcoma tumor PT8DD	GSM528307	Sarcoma tumor PT35DD
GSM528284	Sarcoma tumor PT10DD	GSM528308	Sarcoma tumor PT36DD
GSM528285	Sarcoma tumor PT12DD	GSM528309	Sarcoma tumor PT37DD
GSM528286	Sarcoma tumor PT14DD	GSM528310	Sarcoma tumor PT38DD
GSM528287	Sarcoma tumor PT15DD	GSM528311	Sarcoma tumor PT39DD
GSM528288	Sarcoma tumor PT16DD	GSM528312	Sarcoma tumor PT40DD
GSM528289	Sarcoma tumor PT17DD	GSM528313	Sarcoma tumor PT41DD
GSM528290	Sarcoma tumor PT18DD	GSM528314	Sarcoma tumor PT42DD
GSM528291	Sarcoma tumor PT19DD	GSM528315	Sarcoma tumor PT43DD
GSM528292	Sarcoma tumor PT20DD	GSM528316	Sarcoma tumor PT44DD
GSM528293	Sarcoma tumor PT21DD	GSM528317	Sarcoma tumor PT46DD
GSM528294	Sarcoma tumor PT22DD	GSM528318	Sarcoma tumor PT47DD
GSM528295	Sarcoma tumor PT23DD	GSM528319	Sarcoma tumor PT48DD
GSM528296	Sarcoma tumor PT24DD	GSM528320	Sarcoma tumor PT49DD
GSM528297	Sarcoma tumor PT25DD	GSM528321	Sarcoma tumor PT50DD
GSM528298	Sarcoma tumor PT26DD		
GSM528299	Sarcoma tumor PT27DD		
GSM528300	Sarcoma tumor PT28DD		
GSM528301	Sarcoma tumor PT29DD		
GSM528302	Sarcoma tumor PT30DD		

Table S10: Normal Fat Samples (n=9) used in Transcriptome Profiling from GSE21124

Profiling of Sarcoma Tumors by Array

Barretina J, Taylor BS, Banerji S, Ramos AH et al. Subtype-specific genomic alterations define new targets for soft-tissue sarcoma therapy. *Nat Genet* 2010 Aug;42(8):715-21. PMID: 20601955

Accession URL(s): https://www.cbioportal.org/study/summary?id=sarc_mskcc
https://www.ncbi.nlm.nih.gov/geo/query/acc.cgi?acc=GSE21124

Queried Genes: MDM2, CDK4, GLI1, GLI2

Expression Profiling (GSE21122, CBioPortal)

GEO Sample	Sample Identifier
GSM528425	Control normal fat NF115
GSM528426	Control normal fat NF117
GSM528427	Control normal fat NF119
GSM528428	Control normal fat NF180
GSM528429	Control normal fat NF225
GSM528430	Control normal fat NF84
GSM528431	Control normal fat NF90
GSM528432	Control normal fat NF92
GSM528433	Control normal fat NF95

Table S11: Matched Normal Samples (n=50) used in Genomics Profiling from GSE21124

Profiling of Sarcoma Tumors by Array

Barretina J, Taylor BS, Banerji S, Ramos AH et al. Subtype-specific genomic alterations define new targets for soft-tissue sarcoma therapy. *Nat Genet* 2010 Aug;42(8):715-21. PMID: 20601955

Accession URL(s): https://www.cbioportal.org/study/summary?id=sarc_mskcc
https://www.ncbi.nlm.nih.gov/geo/query/acc.cgi?acc=GSE21124

Queried Genes: MDM2, CDK4, GLI1, GLI2

Genomics Profiling (GSE21123, CBioPortal)

GEO Sample	Sample Identifier	GEO Sample	Sample Identifier
GSM529100	Matched normal for tumor PT1DD	GSM529125	Matched normal for tumor PT26DD
GSM529101	Matched normal for tumor PT2DD	GSM529126	Matched normal for tumor PT27DD
GSM529102	Matched normal for tumor PT3DD	GSM529127	Matched normal for tumor PT28DD
GSM529103	Matched normal for tumor PT4DD	GSM529128	Matched normal for tumor PT29DD
GSM529104	Matched normal for tumor PT5DD	GSM529129	Matched normal for tumor PT30DD
GSM529105	Matched normal for tumor PT6DD	GSM529130	Matched normal for tumor PT31DD
GSM529106	Matched normal for tumor PT7DD	GSM529131	Matched normal for tumor PT32DD
GSM529107	Matched normal for tumor PT8DD	GSM529132	Matched normal for tumor PT33DD
GSM529108	Matched normal for tumor PT9DD	GSM529133	Matched normal for tumor PT34DD
GSM529109	Matched normal for tumor PT10DD	GSM529134	Matched normal for tumor PT35DD
GSM529110	Matched normal for tumor PT11DD	GSM529135	Matched normal for tumor PT36DD
GSM529111	Matched normal for tumor PT12DD	GSM529136	Matched normal for tumor PT37DD
GSM529112	Matched normal for tumor PT13DD	GSM529137	Matched normal for tumor PT38DD
GSM529113	Matched normal for tumor PT14DD	GSM529138	Matched normal for tumor PT39DD
GSM529114	Matched normal for tumor PT15DD	GSM529139	Matched normal for tumor PT40DD
GSM529115	Matched normal for tumor PT16DD	GSM529140	Matched normal for tumor PT41DD
GSM529116	Matched normal for tumor PT17DD	GSM529141	Matched normal for tumor PT42DD
GSM529117	Matched normal for tumor PT18DD	GSM529142	Matched normal for tumor PT43DD
GSM529118	Matched normal for tumor PT19DD	GSM529143	Matched normal for tumor PT44DD
GSM529119	Matched normal for tumor PT20DD	GSM529144	Matched normal for tumor PT45DD
GSM529120	Matched normal for tumor PT21DD	GSM529145	Matched normal for tumor PT46DD
GSM529121	Matched normal for tumor PT22DD	GSM529146	Matched normal for tumor PT47DD
GSM529122	Matched normal for tumor PT23DD	GSM529147	Matched normal for tumor PT48DD
GSM529123	Matched normal for tumor PT24DD	GSM529148	Matched normal for tumor PT49DD
GSM529124	Matched normal for tumor PT25DD	GSM529149	Matched normal for tumor PT50DD

Table S12: Tumor Samples (n=50) used in Genomics Profiling from GSE21124

Profiling of Sarcoma Tumors by Array

Barretina J, Taylor BS, Banerji S, Ramos AH et al. Subtype-specific genomic alterations define new targets for soft-tissue sarcoma therapy. *Nat Genet* 2010 Aug;42(8):715-21. PMID: 20601955

Accession URL(s): https://www.cbioportal.org/study/summary?id=sarc_mskcc
https://www.ncbi.nlm.nih.gov/geo/query/acc.cgi?acc=GSE21124

Queried Genes: MDM2, CDK4, GLI1, GLI2. Genomics Profiling (GSE21123, CBioPortal)

GEO Sample	Sample Identifier	GEO Sample	Sample Identifier
GSM528890	Sarcoma tumor PT1DD	**GSM528919**	Sarcoma tumor PT30DD
GSM528891	Sarcoma tumor PT2DD	GSM528920	Sarcoma tumor PT31DD
GSM528892	Sarcoma tumor PT3DD	GSM528921	Sarcoma tumor PT32DD
GSM528896	Sarcoma tumor PT7DD	GSM528922	Sarcoma tumor PT33DD
GSM528897	Sarcoma tumor PT8DD	GSM528923	Sarcoma tumor PT34DD
GSM528898	Sarcoma tumor PT9DD	GSM528924	Sarcoma tumor PT35DD
GSM528899	Sarcoma tumor PT10DD	GSM528925	Sarcoma tumor PT36DD
GSM528900	Sarcoma tumor PT11DD	GSM528926	Sarcoma tumor PT37DD
GSM528901	Sarcoma tumor PT12DD	GSM528927	Sarcoma tumor PT38DD
GSM528902	Sarcoma tumor PT13DD	GSM528928	Sarcoma tumor PT39DD
GSM528903	Sarcoma tumor PT14DD	GSM528929	Sarcoma tumor PT40DD
GSM528904	Sarcoma tumor PT15DD	GSM528930	Sarcoma tumor PT41DD
GSM528905	Sarcoma tumor PT16DD	GSM528931	Sarcoma tumor PT42DD
GSM528906	Sarcoma tumor PT17DD	GSM528932	Sarcoma tumor PT43DD
GSM528907	Sarcoma tumor PT18DD	GSM528933	Sarcoma tumor PT44DD
GSM528908	Sarcoma tumor PT19DD	GSM528934	Sarcoma tumor PT45DD
GSM528909	Sarcoma tumor PT20DD	GSM528935	Sarcoma tumor PT46DD
GSM528910	Sarcoma tumor PT21DD	GSM528936	Sarcoma tumor PT47DD
GSM528911	Sarcoma tumor PT22DD	GSM528937	Sarcoma tumor PT48DD
GSM528912	Sarcoma tumor PT23DD	GSM528938	Sarcoma tumor PT49DD
GSM528913	Sarcoma tumor PT24DD	GSM528939	Sarcoma tumor PT50DD
GSM528914	Sarcoma tumor PT25DD		
GSM528915	Sarcoma tumor PT26DD		
GSM528916	Sarcoma tumor PT27DD		
GSM528917	Sarcoma tumor PT28DD		
GSM528918	Sarcoma tumor PT29DD		

Table S13: Genomic Alterations in Tumor Samples (n=50) used in Genomics Profiling from GSE21124

Profiling of Sarcoma Tumors by Array

Barretina J, Taylor BS, Banerji S, Ramos AH et al. Subtype-specific genomic alterations define new targets for soft-tissue sarcoma therapy. *Nat Genet* 2010 Aug;42(8):715-21. PMID: 20601955

Accession URL(s): https://www.cbioportal.org/study/summary?id=sarc_mskcc
https://www.ncbi.nlm.nih.gov/geo/query/acc.cgi?acc=GSE21124

Queried Genes: MDM2, CDK4, GLI1, GLI2

Genomics Profiling (GSE21123, CBioPortal)

Study ID	Patient ID	MDM2	CDK4	GLI1	GLI2
sarc_mskcc	PT10DD	AMP (driver)	AMP (driver)	no alteration	no alteration
sarc_mskcc	PT11DD	AMP (driver)	AMP (driver)	no alteration	no alteration
sarc_mskcc	PT12DD	AMP (driver)	AMP (driver)	no alteration	no alteration
sarc_mskcc	PT13DD	no alteration	no alteration	no alteration	AMP
sarc_mskcc	PT14DD	AMP (driver)	no alteration	no alteration	no alteration
sarc_mskcc	PT15DD	AMP (driver)	AMP (driver)	no alteration	no alteration
sarc_mskcc	PT16DD	AMP (driver)	AMP (driver)	no alteration	no alteration
sarc_mskcc	PT17DD	AMP (driver)	AMP (driver)	no alteration	no alteration
sarc_mskcc	PT18DD	AMP (driver)	AMP (driver)	no alteration	no alteration
sarc_mskcc	PT19DD	AMP (driver)	AMP (driver)	no alteration	no alteration
sarc_mskcc	PT1DD	AMP (driver)	AMP (driver)	AMP (driver)	no alteration
sarc_mskcc	PT22DD	AMP (driver)	AMP (driver)	no alteration	no alteration
sarc_mskcc	PT23DD	AMP (driver)	AMP (driver)	no alteration	no alteration
sarc_mskcc	PT25DD	AMP (driver)	AMP (driver)	AMP (driver)	no alteration
sarc_mskcc	PT26DD	AMP (driver)	AMP (driver)	no alteration	no alteration
sarc_mskcc	PT27DD	AMP (driver)	AMP (driver)	AMP (driver)	no alteration
sarc_mskcc	PT28DD	AMP (driver)	AMP (driver)	AMP (driver)	no alteration
sarc_mskcc	PT29DD	AMP (driver)	AMP (driver)	no alteration	no alteration
sarc_mskcc	PT2DD	AMP (driver)	AMP (driver)	no alteration	no alteration
sarc_mskcc	PT30DD	AMP (driver)	AMP (driver)	no alteration	no alteration
sarc_mskcc	PT31DD	AMP (driver)	AMP (driver)	no alteration	no alteration
sarc_mskcc	PT32DD	AMP (driver)	AMP (driver)	no alteration	no alteration
sarc_mskcc	PT33DD	AMP (driver)	AMP (driver)	no alteration	no alteration
sarc_mskcc	PT34DD	AMP (driver)	AMP (driver)	no alteration	no alteration
sarc_mskcc	PT35DD	AMP (driver)	AMP (driver)	AMP (driver)	no alteration
sarc_mskcc	PT36DD	AMP (driver)	AMP (driver)	no alteration	no alteration
sarc_mskcc	PT37DD	AMP (driver)	AMP (driver)	no alteration	no alteration
sarc_mskcc	PT38DD	AMP (driver)	AMP (driver)	no alteration	no alteration
sarc_mskcc	PT39DD	AMP (driver)	AMP (driver)	AMP (driver)	no alteration
sarc_mskcc	PT3DD	AMP (driver)	AMP (driver)	no alteration	no alteration
sarc_mskcc	PT40DD	AMP (driver)	AMP (driver)	AMP (driver)	no alteration
sarc_mskcc	PT41DD	AMP (driver)	AMP (driver)	no alteration	no alteration
sarc_mskcc	PT42DD	AMP (driver)	AMP (driver)	AMP (driver)	no alteration
sarc_mskcc	PT43DD	AMP (driver)	AMP (driver)	no alteration	no alteration
sarc_mskcc	PT44DD	AMP (driver)	AMP (driver)	AMP (driver)	no alteration

sarc_mskcc	PT45DD	AMP (driver)	AMP (driver)	no alteration	no alteration
sarc_mskcc	PT46DD	AMP (driver)	AMP (driver)	AMP (driver)	no alteration
sarc_mskcc	PT48DD	AMP (driver)	AMP (driver)	no alteration	no alteration
sarc_mskcc	PT49DD	AMP (driver)	AMP (driver)	no alteration	no alteration
sarc_mskcc	PT4DD	AMP (driver)	AMP (driver)	AMP (driver)	no alteration
sarc_mskcc	PT50DD	AMP (driver)	AMP (driver)	no alteration	no alteration
sarc_mskcc	PT5DD	AMP (driver)	AMP (driver)	no alteration	no alteration
sarc_mskcc	PT6DD	AMP (driver)	AMP (driver)	AMP (driver)	no alteration
sarc_mskcc	PT7DD	AMP (driver)	AMP (driver)	no alteration	no alteration
sarc_mskcc	PT8DD	AMP (driver)	AMP (driver)	no alteration	no alteration
sarc_mskcc	PT9DD	AMP (driver)	AMP (driver)	no alteration	no alteration
sarc_mskcc	PT20DD	no alteration	no alteration	no alteration	no alteration
sarc_mskcc	PT21DD	no alteration	no alteration	no alteration	no alteration
sarc_mskcc	PT24DD	no alteration	no alteration	no alteration	no alteration
sarc_mskcc	PT47DD	no alteration	no alteration	no alteration	no alteration

Table S14: KEGG Hedgehog pathway Gene Set (hsa04340)

https://www.gsea-msigdb.org/gsea/msigdb/human/geneset/KEGG_HEDGEHOG_SIGNALING_PATHWAY.html?keywords=hedgehog

Gene	Description
BMP2	Bone morphogenetic protein 2; Induces cartilage and bone formation. Stimulates the differentiation of myoblasts into osteoblasts via the EIF2AK3-EIF2A- ATF4 pathway. BMP2 activation of EIF2AK3 stimulates phosphorylation of EIF2A which leads to increased expression of ATF4 which plays a central role in osteoblast differentiation. In addition stimulates TMEM119, which upregulates the expression of ATF4; Belongs to the TGF-beta family
BMP4	Bone morphogenetic protein 4; Induces cartilage and bone formation. Also act in mesoderm induction, tooth development, limb formation and fracture repair. Acts in concert with PTHLH/PTHRP to stimulate ductal outgrowth during embryonic mammary development and to inhibit hair follicle induction (By similarity); Bone morphogenetic proteins
BMP5	Bone morphogenetic protein 5; Induces cartilage and bone formation; Bone morphogenetic proteins
BMP6	Bone morphogenetic protein 6; Induces cartilage and bone formation; Bone morphogenetic proteins
BMP7	Bone morphogenetic protein 7; Induces cartilage and bone formation. May be the osteoinductive factor responsible for the phenomenon of epithelial osteogenesis. Plays a role in calcium regulation and bone homeostasis; Bone morphogenetic proteins
BMP8A	Bone morphogenetic protein 8A; Induces cartilage and bone formation. May be the osteoinductive factor responsible for the phenomenon of epithelial osteogenesis. Plays a role in calcium regulation and bone homeostasis (By similarity). Signaling protein involved in regulation of thermogenesis and energy balance. Proposed to increase the peripheral response of brown adipose tissue (BAT) to adrenergic stimulation while acting centrally in the hypothalamus to increase sympathetic output to BAT; Bone morphogenetic proteins
BMP8B	Bone morphogenetic protein 8B; Induces cartilage and bone formation. May be the osteoinductive factor responsible for the phenomenon of epithelial osteogenesis. Plays a role in calcium regulation and bone homeostasis (By similarity); Belongs to the TGF-beta family
BTRC	F-box/WD repeat-containing protein 1A; Substrate recognition component of a SCF (SKP1-CUL1-F- box protein) E3 ubiquitin-protein ligase complex which mediates the ubiquitination and subsequent proteasomal degradation of target proteins. Recognizes and binds to phosphorylated target proteins. SCF(BTRC) mediates the ubiquitination of CTNNB1 and participates in Wnt signaling. SCF(BTRC) mediates the ubiquitination of NFKBIA, NFKBIB and NFKBIE; the degradation frees the associated NFKB1 to translocate into the nucleus and to activate transcription. Ubiquitination of NFKBIA occurs at 'Lys- 21 [...]
CSNK1A1	Casein kinase I isoform alpha; Casein kinases are operationally defined by their preferential utilization of acidic proteins such as caseins as substrates. It can phosphorylate a large number of proteins. Participates in Wnt signaling. Phosphorylates CTNNB1 at 'Ser-45'. May phosphorylate PER1 and PER2. May play a role in segregating chromosomes during mitosis. May play a role in keratin cytoskeleton disassembly and thereby, it may regulate epithelial cell migration
CSNK1A1L	Casein kinase I isoform alpha-like; Casein kinases are operationally defined by their preferential utilization of acidic proteins such as caseins as substrates. It can phosphorylate a large number of proteins. Participates in Wnt signaling (By similarity)
CSNK1D	Casein kinase I isoform delta; Essential serine/threonine-protein kinase that regulates diverse cellular growth and survival processes including Wnt signaling, DNA repair and circadian rhythms. It can phosphorylate a large number of proteins. Casein kinases are operationally defined by their preferential utilization of acidic proteins such as caseins as substrates. Phosphorylates connexin-43/GJA1, MAP1A, SNAPIN, MAPT/TAU, TOP2A, DCK, HIF1A, EIF6, p53/TP53, DVL2, DVL3, ESR1, AIB1/NCOA3, DNMT1, PKD2, YAP1, PER1 and PER2. Central component of the circadian clock. In balance with PP1, dete [...]
CSNK1E	Casein kinase I isoform epsilon; Casein kinases are operationally defined by their preferential utilization of acidic proteins such as caseins as substrates. Can phosphorylate a large number of proteins. Participates in Wnt signaling. Phosphorylates DVL1 and DVL2. Central component of the circadian clock. In balance with PP1, determines the circadian period length, through the regulation of the speed and rhythmicity of PER1 and PER2 phosphorylation. Controls PER1 and PER2 nuclear transport and degradation. Inhibits cytokine-induced granuloytic differentiation
CSNK1G1	Casein kinase I isoform gamma-1; Serine/threonine-protein kinase. Casein kinases are operationally defined by their preferential utilization of acidic proteins such as caseins as substrates. It can phosphorylate a large number of proteins. Participates in Wnt signaling. Regulates fast synaptic transmission mediated by glutamate (By similarity). Phosphorylates CLSPN

CSNK1G2	Casein kinase I isoform gamma-2; Serine/threonine-protein kinase. Casein kinases are operationally defined by their preferential utilization of acidic proteins such as caseins as substrates. It can phosphorylate a large number of proteins. Participates in Wnt signaling. Phosphorylates COL4A3BP/CERT, MTA1 and SMAD3. Involved in brain development and vesicular trafficking and neurotransmitter releasing from small synaptic vesicles. Regulates fast synaptic transmission mediated by glutamate. SMAD3 phosphorylation promotes its ligand-dependent ubiquitination and subsequent proteasome degra [...]
CSNK1G3	Casein kinase I isoform gamma-3; Serine/threonine-protein kinase. Casein kinases are operationally defined by their preferential utilization of acidic proteins such as caseins as substrates. It can phosphorylate a large number of proteins. Participates in Wnt signaling. Regulates fast synaptic transmission mediated by glutamate (By similarity)
DHH	Desert hedgehog protein; Intercellular signal essential for a variety of patterning events during development. May function as a spermatocyte survival factor in the testes. Essential for testes development; Hedgehog signaling molecule family
FBXW11	F-box/WD repeat-containing protein 11; Substrate recognition component of a SCF (SKP1-CUL1-F- box protein) E3 ubiquitin-protein ligase complex which mediates the ubiquitination and subsequent proteasomal degradation of target proteins. Probably recognizes and binds to phosphorylated target proteins. SCF(FBXW11) mediates the ubiquitination of phosphorylated CTNNB1 and participates in Wnt signaling. SCF(FBXW11) mediates the ubiquitination of phosphorylated NFKBIA, which degradation frees the associated NFKB1 to translocate into the nucleus and to activate transcription. SCF(FBXW11) media [...]
GAS1	Growth arrest-specific protein 1; Specific growth arrest protein involved in growth suppression. Blocks entry to S phase. Prevents cycling of normal and transformed cells
GLI1	Zinc finger protein GLI1; Acts as a transcriptional activator. Binds to the DNA consensus sequence 5'-GACCACCCA-3'. May regulate the transcription of specific genes during normal development. May play a role in craniofacial development and digital development, as well as development of the central nervous system and gastrointestinal tract. Mediates SHH signaling. Plays a role in cell proliferation and differentiation via its role in SHH signaling (Probable); Zinc fingers C2H2-type
GLI2	Zinc finger protein GLI2; Functions as transcription regulator in the hedgehog (Hh) pathway. Functions as transcriptional activator. May also function as transcriptional repressor (By similarity). Requires STK36 for full transcriptional activator activity. Required for normal embryonic development; Zinc fingers C2H2-type
GLI3	Transcriptional activator GLI3; Has a dual function as a transcriptional activator and a repressor of the sonic hedgehog (Shh) pathway, and plays a role in limb development. The full-length GLI3 form (GLI3FL) after phosphorylation and nuclear translocation, acts as an activator (GLI3A) while GLI3R, its C-terminally truncated form, acts as a repressor. A proper balance between the GLI3 activator and the repressor GLI3R, rather than the repressor gradient itself or the activator/repressor ratio gradient, specifies limb digit number and identity. In concert with TRPS1, plays a role in reg [...]
GSK3B	Glycogen synthase kinase-3 beta; Constitutively active protein kinase that acts as a negative regulator in the hormonal control of glucose homeostasis, Wnt signaling and regulation of transcription factors and microtubules, by phosphorylating and inactivating glycogen synthase (GYS1 or GYS2), EIF2B, CTNNB1/beta-catenin, APC, AXIN1, DPYSL2/CRMP2, JUN, NFATC1/NFATC, MAPT/TAU and MACF1. Requires primed phosphorylation of the majority of its substrates. In skeletal muscle, contributes to insulin regulation of glycogen synthesis by phosphorylating and inhibiting GYS1 activity and hence glyc [...]
HHIP	Hedgehog-interacting protein; Modulates hedgehog signaling in several cell types including brain and lung through direct interaction with members of the hedgehog family
IHH	Indian hedgehog protein; Intercellular signal essential for a variety of patterning events during development. Binds to the patched (PTC) receptor, which functions in association with smoothened (SMO), to activate the transcription of target genes. Implicated in endochondral ossification: may regulate the balance between growth and ossification of the developing bones. Induces the expression of parathyroid hormone-related protein (PTHRP) (By similarity); Belongs to the hedgehog family
LRP2	Low-density lipoprotein receptor-related protein 2; Multiligand endocytic receptor (By similarity). Acts together with CUBN to mediate endocytosis of high-density lipoproteins (By similarity). Mediates receptor-mediated uptake of polybasic drugs such as aprotinin, aminoglycosides and polymyxin B (By similarity). In the kidney, mediates the tubular uptake and clearance of leptin (By similarity). Also mediates transport of leptin across the blood-brain barrier through endocytosis at the choroid plexus epithelium (By similarity). Endocytosis of leptin in neuronal cells is required for hyp [...]
PRKACA	cAMP-dependent protein kinase catalytic subunit alpha; Phosphorylates a large number of substrates in the cytoplasm and the nucleus. Regulates the abundance of compartmentalized pools of its regulatory subunits through phosphorylation of PJA2 which binds and ubiquitinates these subunits, leading to their subsequent proteolysis. Phosphorylates CDC25B, ABL1, NFKB1, CLDN3, PSMC5/RPT6, PJA2, RYR2, RORA and VASP. RORA is activated

	by phosphorylation. Required for glucose- mediated adipogenic differentiation increase and osteogenic differentiation inhibition from osteoblasts. Involved in the [...]
PRKACB	cAMP-dependent protein kinase catalytic subunit beta; Mediates cAMP-dependent signaling triggered by receptor binding to GPCRs. PKA activation regulates diverse cellular processes such as cell proliferation, the cell cycle, differentiation and regulation of microtubule dynamics, chromatin condensation and decondensation, nuclear envelope disassembly and reassembly, as well as regulation of intracellular transport mechanisms and ion flux. Regulates the abundance of compartmentalized pools of its regulatory subunits through phosphorylation of PJA2 which binds and ubiquitinates these subu [...]
PRKACG	cAMP-dependent protein kinase catalytic subunit gamma; Phosphorylates a large number of substrates in the cytoplasm and the nucleus; Belongs to the protein kinase superfamily. AGC Ser/Thr protein kinase family. cAMP subfamily
PRKX	cAMP-dependent protein kinase catalytic subunit PRKX; Serine/threonine protein kinase regulated by and mediating cAMP signaling in cells. Acts through phosphorylation of downstream targets that may include CREB, SMAD6 and PKD1 and has multiple functions in cellular differentiation and epithelial morphogenesis. Regulates myeloid cell differentiation through SMAD6 phosphorylation. Involved in nephrogenesis by stimulating renal epithelial cell migration and tubulogenesis. Also involved in angiogenesis through stimulation of endothelial cell proliferation, migration and vascular-like struc [...]
PTCH1	Protein patched homolog 1; Acts as a receptor for sonic hedgehog (SHH), indian hedgehog (IHH) and desert hedgehog (DHH). Associates with the smoothened protein (SMO) to transduce the hedgehog's proteins signal. Seems to have a tumor suppressor function, as inactivation of this protein is probably a necessary, if not sufficient step for tumorigenesis; Belongs to the patched family
PTCH2	Protein patched homolog 2; May have a role in epidermal development. May act as a receptor for Sonic hedgehog (SHH)
RAB23	RAB23, member RAS oncogene family; Ras-related protein Rab-23; The small GTPases Rab are key regulators of intracellular membrane trafficking, from the formation of transport vesicles to their fusion with membranes. Rabs cycle between an inactive GDP-bound form and an active GTP-bound form that is able to recruit to membranes different set of downstream effectors directly responsible for vesicle formation, movement, tethering and fusion. Together with SUFU, prevents nuclear import of GLI1, and thereby inhibits GLI1 transcription factor activity. Regulates GLI1 in differentiating chondr [...]
SHH	Sonic hedgehog protein; Sonic hedgehog protein: The C-terminal part of the sonic hedgehog protein precursor displays an autoproteolysis and a cholesterol transferase activity (By similarity). Both activities result in the cleavage of the full-length protein into two parts (ShhN and ShhC) followed by the covalent attachment of a cholesterol moiety to the C-terminal of the newly generated ShhN (By similarity). Both activities occur in the reticulum endoplasmic (By similarity). Once cleaved, ShhC is degraded in the endoplasmic reticulum (By similarity); Hedgehog signaling molecule family
SMO	Smoothened, frizzled class receptor; Smoothened homolog; G protein-coupled receptor that probably associates with the patched protein (PTCH) to transduce the hedgehog's proteins signal. Binding of sonic hedgehog (SHH) to its receptor patched is thought to prevent normal inhibition by patched of smoothened (SMO). Required for the accumulation of KIF7, GLI2 and GLI3 in the cilia. Interacts with DLG5 at the ciliary base to induce the accumulation of KIF7 and GLI2 at the ciliary tip for GLI2 activation (By similarity)
STK36	Serine/threonine-protein kinase 36; Serine/threonine protein kinase which plays an important role in the sonic hedgehog (Shh) pathway by regulating the activity of GLI transcription factors. Controls the activity of the transcriptional regulators GLI1, GLI2 and GLI3 by opposing the effect of SUFU and promoting their nuclear localization. GLI2 requires an additional function of STK36 to become transcriptionally active, but the enzyme does not need to possess an active kinase catalytic site for this to occur. Required for postnatal development, possibly by regulating the homeostasis of c [...]
SUFU	Suppressor of fused homolog; Negative regulator in the hedgehog signaling pathway. Down-regulates GLI1-mediated transactivation of target genes. Down- regulates GLI2-mediated transactivation of target genes. Part of a corepressor complex that acts on DNA-bound GLI1. May also act by linking GLI1 to BTRC and thereby targeting GLI1 to degradation by the proteasome. Sequesters GLI1, GLI2 and GLI3 in the cytoplasm, this effect is overcome by binding of STK36 to both SUFU and a GLI protein. Negative regulator of beta- catenin signaling. Regulates the formation of either the repressor form (G [...]
WNT1	Wingless-type mmtv integration site family, member 1; Proto-oncogene Wnt-1; Ligand for members of the frizzled family of seven transmembrane receptors. Acts in the canonical Wnt signaling pathway by promoting beta-catenin-dependent transcriptional activation. In some developmental processes, is also a ligand for the coreceptor RYK, thus triggering Wnt signaling. Probable developmental protein. May be a signaling molecule important in CNS development. Is likely to signal over only few cell diameters. Has a role in osteoblast function and bone development

WNT10A	Wingless-type mmtv integration site family, member 10; Protein Wnt-10a; Ligand for members of the frizzled family of seven transmembrane receptors. Probable developmental protein. May be a signaling molecule important in CNS development. Is likely to signal over only few cell diameters; Belongs to the Wnt family
WNT10B	Wingless-type mmtv integration site family, member 10; Protein Wnt-10b; Member of the Wnt ligand gene family that encodes for secreted proteins, which activate the Wnt signaling cascade. Specifically activates canonical Wnt/beta-catenin signaling and thus triggers beta-catenin/LEF/TCF-mediated transcriptional programs. Involved in signaling networks controlling stemness, pluripotency and cell fate decisions. Acts in the immune system, mammary gland, adipose tissue, bone and skin; Belongs to the Wnt family
WNT11	Wingless-type mmtv integration site family, member 11; Protein Wnt-11; Ligand for members of the frizzled family of seven transmembrane receptors. Probable developmental protein. May be a signaling molecule which affects the development of discrete regions of tissues. Is likely to signal over only few cell diameters
WNT16	Wingless-type mmtv integration site family, member 16; Protein Wnt-16; Ligand for members of the frizzled family of seven transmembrane receptors. Probable developmental protein. May be a signaling molecule which affects the development of discrete regions of tissues. Is likely to signal over only few cell diameters (By similarity)
WNT2	Wingless-type mmtv integration site family, member 2; Protein Wnt-2; Ligand for members of the frizzled family of seven transmembrane receptors. Probable developmental protein. May be a signaling molecule which affects the development of discrete regions of tissues. Is likely to signal over only few cell diameters; Belongs to the Wnt family
WNT2B	Wingless-type mmtv integration site family, member 2; Protein Wnt-2b; Ligand for members of the frizzled family of seven transmembrane receptors. Probable developmental protein. May be a signaling molecule which affects the development of discrete regions of tissues. Is likely to signal over only few cell diameters. May be involved in normal development or differentiation as well as in carcinogenesis
WNT3	Wingless-type mmtv integration site family, member 3; Proto-oncogene Wnt-3; Ligand for members of the frizzled family of seven transmembrane receptors. Wnt-3 and Wnt-3a play distinct roles in cell-cell signaling during morphogenesis of the developing neural tube (By similarity)
WNT3A	Wingless-type mmtv integration site family, member 3; Protein Wnt-3a; Ligand for members of the frizzled family of seven transmembrane receptors. Wnt-3 and Wnt-3a play distinct roles in cell-cell signaling during morphogenesis of the developing neural tube; Endogenous ligands
WNT4	Wingless-type mmtv integration site family, member 4; Protein Wnt-4; Ligand for members of the frizzled family of seven transmembrane receptors. Probable developmental protein. May be a signaling molecule which affects the development of discrete regions of tissues. Is likely to signal over only few cell diameters (By similarity). Overexpression may be associated with abnormal proliferation in human breast tissue; Endogenous ligands
WNT5A	Wingless-type mmtv integration site family, member 5; Protein Wnt-5a; Ligand for members of the frizzled family of seven transmembrane receptors. Can activate or inhibit canonical Wnt signaling, depending on receptor context. In the presence of FZD4, activates beta-catenin signaling. In the presence of ROR2, inhibits the canonical Wnt pathway by promoting beta-catenin degradation through a GSK3-independent pathway which involves down-regulation of beta-catenin-induced reporter gene expression. Suppression of the canonical pathway allows chondrogenesis to occur and inhibits tumor format [...]
WNT5B	Wingless-type mmtv integration site family, member 5; Protein Wnt-5b; Ligand for members of the frizzled family of seven transmembrane receptors. Probable developmental protein. May be a signaling molecule which affects the development of discrete regions of tissues. Is likely to signal over only few cell diameters (By similarity); Belongs to the Wnt family
WNT6	Wingless-type mmtv integration site family, member 6; Protein Wnt-6; Ligand for members of the frizzled family of seven transmembrane receptors. Probable developmental protein. May be a signaling molecule which affects the development of discrete regions of tissues. Is likely to signal over only few cell diameters. Together with CAV1 may promote chemoresistance of gastric cancer cells to DNA-damaging anthracycline drugs through the activation of the canonical Wnt receptor signaling pathway
WNT7A	Wingless-type mmtv integration site family, member 7; Protein Wnt-7a; Ligand for members of the frizzled family of seven transmembrane receptors. Probable developmental protein. Signaling by Wnt-7a allows sexually dimorphic development of the mullerian ducts (By similarity)
WNT7B	Wingless-type mmtv integration site family, member 7; Protein Wnt-7b; Ligand for members of the frizzled family of seven transmembrane receptors. Probable developmental protein. May be a signaling molecule which affects the development of discrete regions of tissues. Is likely to signal over only few cell diameters (By similarity); Endogenous ligands
WNT8A	Wingless-type mmtv integration site family, member 8; Protein Wnt-8a; Ligand for members of the frizzled family of seven transmembrane receptors. May play an important role in the development and differentiation of certain forebrain structures, notably the hippocampus

WNT8B	Wingless-type mmtv integration site family, member 8; Protein Wnt-8b; Ligand for members of the frizzled family of seven transmembrane receptors. May play an important role in the development and differentiation of certain forebrain structures, notably the hippocampus
WNT9A	Wingless-type mmtv integration site family, member 9; Protein Wnt-9a; Ligand for members of the frizzled family of seven transmembrane receptors. Probable developmental protein. May be a signaling molecule which affects the development of discrete regions of tissues. Is likely to signal over only few cell diameters (By similarity)
WNT9B	Wingless-type mmtv integration site family, member 9; Protein Wnt-9b; Ligand for members of the frizzled family of seven transmembrane receptors. Probable developmental protein. May be a signaling molecule which affects the development of discrete regions of tissues. Is likely to signal over only few cell diameters (By similarity)
ZIC2	Zinc finger protein ZIC 2; Acts as a transcriptional activator or repressor. Plays important roles in the early stage of organogenesis of the CNS. Activates the transcription of the serotonin transporter SERT in uncrossed ipsilateral retinal ganglion cells (iRGCs) to refine eye-specific projections in primary visual targets. Its transcriptional activity is repressed by MDFIC. Involved in the formation of the ipsilateral retinal projection at the optic chiasm midline. Drives the expression of EPHB1 on ipsilaterally projecting growth cones. Binds to the minimal GLI-consensus sequence 5'- [...]

Supplemental Table S15: Gene Set GCNP_SHH_UP_EARLY.V1_UP

https://www.gsea-msigdb.org/gsea/msigdb/cards/GCNP_SHH_UP_EARLY.V1_UP

Source Id	NCBI (Entrez) Gene Id	Gene Symbol	Gene Description
0610005C13Rik			
1100001G20Rik	645638	WFDC21P	WAP four-disulfide core domain 2...
1190002H23Rik	28984	RGCC	regulator of cell cycle [Source:...
1810030O07Rik	159013	CXorf38	chromosome X open reading frame ...
2410003P15Rik	55245	UQCC1	ubiquinol-cytochrome c reductase...
2510039O18Rik	90231	KIAA2013	KIAA2013 [Source:HGNC Symbol;Acc...
2610003J06Rik	339123	JMJD8	jumonji domain containing 8 [Sou...
2610201A13Rik			
2810453I06Rik	84792	FAM220A	family with sequence similarity ...
3300001P08Rik	51747	LUC7L3	LUC7 like 3 pre-mRNA splicing fa...
4732407F15Rik	353288	KRT26	keratin 26 [Source:HGNC Symbol;A...
4930517K11Rik	116832	RPL39L	ribosomal protein L39 like [Sour...
4933426K21Rik	57494	RIMKLB	ribosomal modification protein r...
5830411E10Rik	64859	NABP1	nucleic acid binding protein 1 [...
AA408278	79939	SLC35E1	solute carrier family 35 member ...
ABCB10	23456	ABCB10	ATP binding cassette subfamily B...
ADAMTS1	9510	ADAMTS1	ADAM metallopeptidase with throm...
ADCY6	112	ADCY6	adenylate cyclase 6 [Source:HGNC...
AFF4	27125	AFF4	ALF transcription elongation fac...
AI931714	90826	PRMT9	protein arginine methyltransfera...
ANKRD13C	81573	ANKRD13C	ankyrin repeat domain 13C [Sourc...
ANLN	54443	ANLN	anillin, actin binding protein [...
APRIN	23047	PDS5B	PDS5 cohesin associated factor B...
ARHGAP8	23779	ARHGAP8	Rho GTPase activating protein 8 ...
ARHGEF1	9138	ARHGEF1	Rho guanine nucleotide exchange ...
ARL5A	26225	ARL5A	ADP ribosylation factor like GTP...
ARL6IP2	64225	ATL2	atlastin GTPase 2 [Source:HGNC S...

ATF1	466	ATF1	activating transcription factor ...
AURKA	6790	AURKA	aurora kinase A [Source:HGNC Sym...
AURKB	9212	AURKB	aurora kinase B [Source:HGNC Sym...
AZIN1	51582	AZIN1	antizyme inhibitor 1 [Source:HGN...
B930007L02Rik	9730	DCAF1	DDB1 and CUL4 associated factor ...
BCKDHA	593	BCKDHA	branched chain keto acid dehydro...
BEXL1	56271	BEX4	brain expressed X-linked 4 [Sour...
C330006A16Rik	90120	TMEM250	transmembrane protein 250 [Sourc...
C330011F01Rik // / 64			
C76434			
C77808			
C80865			
CCDC99	54908	SPDL1	spindle apparatus coiled-coil pr...
CCNB1-RS1 /// LO C231			
CCNJ	54619	CCNJ	cyclin J [Source:HGNC Symbol;Acc...
CD276	80381	CD276	CD276 molecule [Source:HGNC Symb...
CD2BP2	10421	CD2BP2	CD2 cytoplasmic tail binding pro...
CD55	1604	CD55	CD55 molecule (Cromer blood grou...
CDC23	8697	CDC23	cell division cycle 23 [Source:H...
CHEK1	1111	CHEK1	checkpoint kinase 1 [Source:HGNC...
CKS2	1164	CKS2	CDC28 protein kinase regulatory ...
CLCN6	1185	CLCN6	chloride voltage-gated channel 6...
CNOT2	4848	CNOT2	CCR4-NOT transcription complex s...
COX7B	1349	COX7B	cytochrome c oxidase subunit 7B ...
CR2	1380	CR2	complement C3d receptor 2 [Sourc...
CSF1	1435	CSF1	colony stimulating factor 1 [Sou...
Cyp2c29	1557	CYP2C19	cytochrome P450 family 2 subfami...
D11WSU99E			
D17H6S56E-5			
D3Wsu161e	51574	LARP7	La ribonucleoprotein 7, transcri...
D7ERTD183E			
D930048N14Rik			

DARS	1615	DARS1	aspartyl-tRNA synthetase 1 [Sour...
DCPS	28960	DCPS	decapping enzyme, scavenger [Sou...
DHFR	1719	DHFR	dihydrofolate reductase [Source:...
DHODH	1723	DHODH	dihydroorotate dehydrogenase (qu...
DIO1	1733	DIO1	iodothyronine deiodinase 1 [Sour...
DNAJC3	5611	DNAJC3	DnaJ heat shock protein family (...
DOLPP1	57171	DOLPP1	dolichyldiphosphatase 1 [Source:...
E230006M18Rik			
E2F3	1871	E2F3	E2F transcription factor 3 [Sour...
E2F8	79733	E2F8	E2F transcription factor 8 [Sour...
ELOVL2	54898	ELOVL2	ELOVL fatty acid elongase 2 [Sou...
Epb4.1l4b	54566	EPB41L4B	erythrocyte membrane protein ban...
FBXO45	200933	FBXO45	F-box protein 45 [Source:HGNC Sy...
FCGRT	2217	FCGRT	Fc gamma receptor and transporte...
FEN1	2237	FEN1	flap structure-specific endonucl...
FIP1L1	81608	FIP1L1	factor interacting with PAPOLA a...
FNBP4	23360	FNBP4	formin binding protein 4 [Source...
FNTB	2342	FNTB	farnesyltransferase, CAAX box, b...
FOSL1	8061	FOSL1	FOS like 1, AP-1 transcription f...
FOXK1	221937	FOXK1	forkhead box K1 [Source:HGNC Sym...
FTSJ3	117246	FTSJ3	FtsJ RNA 2'-O-methyltransferase ...
FUSIP1	10772	SRSF10	serine and arginine rich splicin...
GGPS1	9453	GGPS1	geranylgeranyl diphosphate synth...
GNL3	26354	GNL3	G protein nucleolar 3 [Source:HG...
GPATC2	55105	GPATCH2	G-patch domain containing 2 [Sou...
GTF2H1	2965	GTF2H1	general transcription factor IIH...
H2-Q8	3136	HLA-H	major histocompatibility complex...
HDGFL1	154150	HDGFL1	HDGF like 1 [Source:HGNC Symbol;...
HMGCL	3155	HMGCL	3-hydroxy-3-methylglutaryl-CoA l...

ICAM5	7087	ICAM5	intercellular adhesion molecule ...
INTS7	25896	INTS7	integrator complex subunit 7 [So...
IRF5	3663	IRF5	interferon regulatory factor 5 [...
IRF6	3664	IRF6	interferon regulatory factor 6 [...
KBTBD7	84078	KBTBD7	kelch repeat and BTB domain cont...
KCNJ4	3761	KCNJ4	potassium inwardly rectifying ch...
KIF1A	547	KIF1A	kinesin family member 1A [Source...
KIF1B	23095	KIF1B	kinesin family member 1B [Source...
KIF4A	24137	KIF4A	kinesin family member 4A [Source...
KIFC1	3833	KIFC1	kinesin family member C1 [Source...
KPNA2 /// LOC670 551			
KRT1-12			
KRTAP6-3	337968	KRTAP6-3	keratin associated protein 6-3 [...
LATS1	9113	LATS1	large tumor suppressor kinase 1 ...
LBR	3930	LBR	lamin B receptor [Source:HGNC Sy...
LOC382653	28412	IGHV3-66	immunoglobulin heavy variable 3-...
LOC435970	7562	ZNF708	zinc finger protein 708 [Source:...
LOC435970 /// LO C668			
LOC541456			
LOXL2	4017	LOXL2	lysyl oxidase like 2 [Source:HGN...
LUC7L2	51631	LUC7L2	LUC7 like 2, pre-mRNA splicing f...
MAP4K3 /// LOC67 5560			
MAPK14	1432	MAPK14	mitogen-activated protein kinase...
MATR3	9782	MATR3	matrin 3 [Source:HGNC Symbol;Acc...
MBL2	4153	MBL2	mannose binding lectin 2 [Source...
MBTD1	54799	MBTD1	mbt domain containing 1 [Source:...
MCM10	55388	MCM10	minichromosome maintenance 10 re...
MCM5	4174	MCM5	minichromosome maintenance compl...
MCM6	4175	MCM6	minichromosome maintenance compl...
MCPT4			

MLLT10	8028	MLLT10	MLLT10 histone lysine methyltran...
MNAB	54542	RC3H2	ring finger and CCCH-type domain...
MP4			
MPDZ	8777	MPDZ	multiple PDZ domain crumbs cell ...
MRE11A	4361	MRE11	MRE11 homolog, double strand bre...
MRPL50	54534	MRPL50	mitochondrial ribosomal protein ...
MRPL9	65005	MRPL9	mitochondrial ribosomal protein ...
MSH6	2956	MSH6	mutS homolog 6 [Source:HGNC Symb...
MT4	84560	MT4	metallothionein 4 [Source:HGNC S...
MTA1	9112	MTA1	metastasis associated 1 [Source:...
MYB	4602	MYB	MYB proto-oncogene, transcriptio...
MYT1L	23040	MYT1L	myelin transcription factor 1 li...
Man1a	4121	MAN1A1	mannosidase alpha class 1A membe...
Muc10			
NAP1L1	4673	NAP1L1	nucleosome assembly protein 1 li...
NCAM2	4685	NCAM2	neural cell adhesion molecule 2 ...
NEK9	91754	NEK9	NIMA related kinase 9 [Source:HG...
NR0B1	190	NR0B1	nuclear receptor subfamily 0 gro...
NTHL1	4913	NTHL1	nth like DNA glycosylase 1 [Sour...
NUP107	57122	NUP107	nucleoporin 107 [Source:HGNC Sym...
NVL	4931	NVL	nuclear VCP like [Source:HGNC Sy...
OVGP1	5016	OVGP1	oviductal glycoprotein 1 [Source...
OXNAD1	92106	OXNAD1	oxidoreductase NAD binding domai...
OXSM	54995	OXSM	3-oxoacyl-ACP synthase, mitochon...
PARG	8505	PARG	poly(ADP-ribose) glycohydrolase ...
PHTF2	57157	PHTF2	putative homeodomain transcripti...
PLCG2	5336	PLCG2	phospholipase C gamma 2 [Source:...
POLA2	23649	POLA2	DNA polymerase alpha 2, accessor...
PPP1CB	5500	PPP1CB	protein phosphatase 1 catalytic ...
PRC1	9055	PRC1	protein regulator of cytokinesis...

PSMA4 /// LOC670 627			
PUM2	23369	PUM2	pumilio RNA binding family membe...
RBBP6	5930	RBBP6	RB binding protein 6, ubiquitin ...
RBM39	9584	RBM39	RNA binding motif protein 39 [So...
RETN	56729	RETN	resistin [Source:HGNC Symbol;Acc...
RHPN2	85415	RHPN2	rhophilin Rho GTPase binding pro...
RNASEH2A /// LOC 6334			
RNF103	7844	RNF103	ring finger protein 103 [Source:...
RNF12	51132	RLIM	ring finger protein, LIM domain ...
RPL10A /// LOC54 6651			
RPLP2 /// 260000 1A11			
RRM1	6240	RRM1	ribonucleotide reductase catalyt...
RSNL2	79745	CLIP4	CAP-Gly domain containing linker...
RUNX1T1	862	RUNX1T1	RUNX1 partner transcriptional co...
RYK	6259	RYK	receptor like tyrosine kinase [S...
Reg1	5967	REG1A	regenerating family member 1 alp...
SDC3	9672	SDC3	syndecan 3 [Source:HGNC Symbol;A...
SEH1L	81929	SEH1L	SEH1 like nucleoporin [Source:HG...
SFRS1	6426	SRSF1	serine and arginine rich splicin...
SGOL1	151648	SGO1	shugoshin 1 [Source:HGNC Symbol;...
SHC1	6464	SHC1	SHC adaptor protein 1 [Source:HG...
SHE	126669	SHE	Src homology 2 domain containing...
SLC23A2	9962	SLC23A2	solute carrier family 23 member ...
SLC27A1	376497	SLC27A1	solute carrier family 27 member ...
SMC2	10592	SMC2	structural maintenance of chromo...
SNAPC2	6618	SNAPC2	small nuclear RNA activating com...
SNORD22	9304	SNORD22	small nucleolar RNA, C/D box 22 ...
SOX10	6663	SOX10	SRY-box transcription factor 10 ...
STAG2	10735	STAG2	stromal antigen 2 [Source:HGNC S...

STAT5A	6776	STAT5A	signal transducer and activator ...
STRN3	29966	STRN3	striatin 3 [Source:HGNC Symbol;A...
STYX	6815	STYX	serine/threonine/tyrosine intera...
TBP	6908	TBP	TATA-box binding protein [Source...
TIA1	7072	TIA1	TIA1 cytotoxic granule associate...
TINF2	26277	TINF2	TERF1 interacting nuclear factor...
TLOC1	7095	SEC62	SEC62 homolog, preprotein transl...
TMPRSS2	7113	TMPRSS2	transmembrane serine protease 2 ...
TNNC2	7125	TNNC2	troponin C2, fast skeletal type ...
TNPO1	3842	TNPO1	transportin 1 [Source:HGNC Symbo...
TNPO1 /// LOC634263			
TOP2A	7153	TOP2A	DNA topoisomerase II alpha [Sour...
TOP2B	7155	TOP2B	DNA topoisomerase II beta [Sourc...
TRAIP	10293	TRAIP	TRAF interacting protein [Source...
TSHB	7252	TSHB	thyroid stimulating hormone subu...
TYMS	7298	TYMS	thymidylate synthetase [Source:H...
UBE2T	29089	UBE2T	ubiquitin conjugating enzyme E2 ...
UPF3B	65109	UPF3B	UPF3B regulator of nonsense medi...
VPS4B	9525	VPS4B	vacuolar protein sorting 4 homol...
Vhlh	7428	VHL	von Hippel-Lindau tumor suppress...
XPO1	7514	XPO1	exportin 1 [Source:HGNC Symbol;A...
ZFP281			
ZZZ3	26009	ZZZ3	zinc finger ZZ-type containing 3...

Supplemental Table S16: Gene Set GCNP_SHH_UP_LATE.V1_UP

https://www.gsea-msigdb.org/gsea/msigdb/cards/GCNP_SHH_UP_LATE.V1_UP

Source Id	NCBI (Entrez) Gene Id	Gene Symbol	Gene Description
1190017O12Rik	54065	SMIM11	small integral membrane protein...
1500003O03Rik	11261	CHP1	calcineurin like EF-hand protei...
1810013D10Rik	389203	SMIM20	small integral membrane protein...
2210419D22Rik			
2610012O22Rik	51154	MRTO4	MRT4 homolog, ribosome maturati...
2610029G23Rik	51260	PBDC1	polysaccharide biosynthesis dom...
2610205E22Rik	28989	NTMT1	N-terminal Xaa-Pro-Lys N-methyl...
2810422B04Rik	55037	PTCD3	pentatricopeptide repeat domain...
3300001P08Rik	51747	LUC7L3	LUC7 like 3 pre-mRNA splicing f...
A430005L14Rik	339448	C1orf174	chromosome 1 open reading frame...
A930012N16Rik			
ABCE1	6059	ABCE1	ATP binding cassette subfamily ...
AI503316	3192	HNRNPU	heterogeneous nuclear ribonucle...
ARIH1	25820	ARIH1	ariadne RBR E3 ubiquitin protei...
ARL5A	26225	ARL5A	ADP ribosylation factor like GT...
ASB3	51130	ASB3	ankyrin repeat and SOCS box con...
AURKA	6790	AURKA	aurora kinase A [Source:HGNC Sy...
AURKB	9212	AURKB	aurora kinase B [Source:HGNC Sy...
B930007L02Rik	9730	DCAF1	DDB1 and CUL4 associated factor...
BC020002	54468	MIOS	meiosis regulator for oocyte de...
BOK	666	BOK	BCL2 family apoptosis regulator...
C130065N10Rik			
C79407	55320	MIS18BP1	MIS18 binding protein 1 [Source...
CASD1	64921	CASD1	CAS1 domain containing 1 [Sourc...
CASP4	837	CASP4	caspase 4 [Source:HGNC Symbol;A...
CBX3 /// LOC629578 /			
CCNB1-RS1 /// LOC231			

CCND2	894	CCND2	cyclin D2 [Source:HGNC Symbol;A...
CCR2	729230	CCR2	C-C motif chemokine receptor 2 ...
CD5	921	CD5	CD5 molecule [Source:HGNC Symbo...
CD59A			
CD6	923	CD6	CD6 molecule [Source:HGNC Symbo...
CD80	941	CD80	CD80 molecule [Source:HGNC Symb...
CDC25A	993	CDC25A	cell division cycle 25A [Source...
CDC25C	995	CDC25C	cell division cycle 25C [Source...
CDCA7	83879	CDCA7	cell division cycle associated ...
CFDP1	10428	CFDP1	craniofacial development protei...
CHEK1	1111	CHEK1	checkpoint kinase 1 [Source:HGN...
CKS2	1164	CKS2	CDC28 protein kinase regulatory...
CLCN5	1184	CLCN5	chloride voltage-gated channel ...
CLIC4	25932	CLIC4	chloride intracellular channel ...
COIL	8161	COIL	coilin [Source:HGNC Symbol;Acc:...
COPB1	1315	COPB1	COPI coat complex subunit beta ...
COX7B	1349	COX7B	cytochrome c oxidase subunit 7B...
CREM	1390	CREM	cAMP responsive element modulat...
CSPG2	1462	VCAN	versican [Source:HGNC Symbol;Ac...
CUL4A	8451	CUL4A	cullin 4A [Source:HGNC Symbol;A...
CYP4B1	1580	CYP4B1	cytochrome P450 family 4 subfam...
Cdc2a	983	CDK1	cyclin dependent kinase 1 [Sour...
D17H6S56E-5			
D3Wsu161e	51574	LARP7	La ribonucleoprotein 7, transcr...
D530031C13Rik	54454	ATAD2B	ATPase family AAA domain contai...
D9ERTD306E			
DCPP			
DDX39	10212	DDX39A	DExD-box helicase 39A [Source:H...
DDX3X	1654	DDX3X	DEAD-box helicase 3 X-linked [S...
DES	1674	DES	desmin [Source:HGNC Symbol;Acc:...
DIDO1	11083	DIDO1	death inducer-obliterator 1 [So...

DLG7	9787	DLGAP5	DLG associated protein 5 [Sourc...
DNM1L	10059	DNM1L	dynamin 1 like [Source:HGNC Sym...
E2F8	79733	E2F8	E2F transcription factor 8 [Sou...
EIF2A	83939	EIF2A	eukaryotic translation initiati...
EIF3S10	8661	EIF3A	eukaryotic translation initiati...
ELAVL2	1993	ELAVL2	ELAV like RNA binding protein 2...
ELOVL2	54898	ELOVL2	ELOVL fatty acid elongase 2 [So...
FAM60A	58516	SINHCAF	SIN3-HDAC complex associated fa...
FANCC	2176	FANCC	FA complementation group C [Sou...
FANCM	57697	FANCM	FA complementation group M [Sou...
FBXO11	80204	FBXO11	F-box protein 11 [Source:HGNC S...
FMR1NB	158521	FMR1NB	FMR1 neighbor [Source:HGNC Symb...
FOXA1	3169	FOXA1	forkhead box A1 [Source:HGNC Sy...
FRMD6	122786	FRMD6	FERM domain containing 6 [Sourc...
FTSJ3	117246	FTSJ3	FtsJ RNA 2'-O-methyltransferase...
FUSIP1	10772	SRSF10	serine and arginine rich splici...
GALNT3	2591	GALNT3	polypeptide N-acetylgalactosami...
GART	2618	GART	phosphoribosylglycinamide formy...
GBP2	2634	GBP2	guanylate binding protein 2 [So...
GFM2	84340	GFM2	GTP dependent ribosome recyclin...
GNL3	26354	GNL3	G protein nucleolar 3 [Source:H...
HIGD1A	25994	HIGD1A	HIG1 hypoxia inducible domain f...
HNRPA3	10151	HNRNPA3P1	heterogeneous nuclear ribonucle...
HSD3B4			
HSPB2	3316	HSPB2	heat shock protein family B (sm...
IDH2	3418	IDH2	isocitrate dehydrogenase (NADP(...
IFNA11			
IGF2	3481	IGF2	insulin like growth factor 2 [S...
INCENP	3619	INCENP	inner centromere protein [Sourc...
IVNS1ABP	10625	IVNS1ABP	influenza virus NS1A binding pr...

KIF2C	11004	KIF2C	kinesin family member 2C [Sourc...
KIFC1	3833	KIFC1	kinesin family member C1 [Sourc...
KLRA6			
KLRA8			
KRTAP6-3	337968	KRTAP6-3	keratin associated protein 6-3 ...
LBR	3930	LBR	lamin B receptor [Source:HGNC S...
LEFTY1	10637	LEFTY1	left-right determination factor...
LIG1	3978	LIG1	DNA ligase 1 [Source:HGNC Symbo...
LLGL2	3993	LLGL2	LLGL scribble cell polarity com...
LOC441376	441376	AARD	alanine and arginine rich domai...
LOC665775 /// LO C675			
LOC671598			
LOXL2	4017	LOXL2	lysyl oxidase like 2 [Source:HG...
LRRC23	10233	LRRC23	leucine rich repeat containing ...
MAGOH	4116	MAGOH	mago homolog, exon junction com...
MAP3K7	6885	MAP3K7	mitogen-activated protein kinas...
MATR3	9782	MATR3	matrin 3 [Source:HGNC Symbol;Ac...
MBD2	8932	MBD2	methyl-CpG binding domain prote...
MCM10	55388	MCM10	minichromosome maintenance 10 r...
MCM4	4173	MCM4	minichromosome maintenance comp...
MCM5	4174	MCM5	minichromosome maintenance comp...
MCM6	4175	MCM6	minichromosome maintenance comp...
MDN1	23195	MDN1	midasin AAA ATPase 1 [Source:HG...
METAP1	23173	METAP1	methionyl aminopeptidase 1 [Sou...
MOBK1B	55233	MOB1A	MOB kinase activator 1A [Source...
MPHOSPH10	10199	MPHOSPH10	M-phase phosphoprotein 10 [Sour...
MRPL18	29074	MRPL18	mitochondrial ribosomal protein...
MUC1	4582	MUC1	mucin 1, cell surface associate...
MYB	4602	MYB	MYB proto-oncogene, transcripti...
NARG1	80155	NAA15	N-alpha-acetyltransferase 15, N...

NAT12	122830	NAA30	N-alpha-acetyltransferase 30, N...
NKX2-2	4821	NKX2-2	NK2 homeobox 2 [Source:HGNC Sym...
NOL5A	10528	NOP56	NOP56 ribonucleoprotein [Source...
NPAS1	4861	NPAS1	neuronal PAS domain protein 1 [...
NPM1	4869	NPM1	nucleophosmin 1 [Source:HGNC Sy...
NPTX1	4884	NPTX1	neuronal pentraxin 1 [Source:HG...
NR5A2	2494	NR5A2	nuclear receptor subfamily 5 gr...
NUSAP1	51203	NUSAP1	nucleolar and spindle associate...
NXF1	10482	NXF1	nuclear RNA export factor 1 [So...
P2RY2	5029	P2RY2	purinergic receptor P2Y2 [Sourc...
PCNT	5116	PCNT	pericentrin [Source:HGNC Symbol...
PCSK2	5126	PCSK2	proprotein convertase subtilisi...
PDCD4 /// LOC670 861			
PFDN6	10471	PFDN6	prefoldin subunit 6 [Source:HGN...
PHTF2	57157	PHTF2	putative homeodomain transcript...
PIK3CA	5290	PIK3CA	phosphatidylinositol-4,5-bispho...
PLEKHA7	144100	PLEKHA7	pleckstrin homology domain cont...
PLK2	10769	PLK2	polo like kinase 2 [Source:HGNC...
POLA2	23649	POLA2	DNA polymerase alpha 2, accesso...
POLRMT	5442	POLRMT	RNA polymerase mitochondrial [S...
PRC1	9055	PRC1	protein regulator of cytokinesi...
PSMC3IP	29893	PSMC3IP	PSMC3 interacting protein [Sour...
PSME4	23198	PSME4	proteasome activator subunit 4 ...
PTPRC	5788	PTPRC	protein tyrosine phosphatase re...
PTPRCAP	5790	PTPRCAP	protein tyrosine phosphatase re...
PVRL2	5819	NECTIN2	nectin cell adhesion molecule 2...
RAG2	5897	RAG2	recombination activating 2 [Sou...
RNF12	51132	RLIM	ring finger protein, LIM domain...
RRM1	6240	RRM1	ribonucleotide reductase cataly...

RRM2	6241	RRM2	ribonucleotide reductase regula...
RSNL2	79745	CLIP4	CAP-Gly domain containing linke...
RYK	6259	RYK	receptor like tyrosine kinase [...
SACM1L	22908	SACM1L	SAC1 like phosphatidylinositide...
SARDH	1757	SARDH	sarcosine dehydrogenase [Source...
SDC2	6383	SDC2	syndecan 2 [Source:HGNC Symbol;...
SERPINB9	5272	SERPINB9	serpin family B member 9 [Sourc...
SFRP1	6422	SFRP1	secreted frizzled related prote...
SFRS1	6426	SRSF1	serine and arginine rich splici...
SGOL1	151648	SGO1	shugoshin 1 [Source:HGNC Symbol...
SIX4	51804	SIX4	SIX homeobox 4 [Source:HGNC Sym...
SLC12A1	6557	SLC12A1	solute carrier family 12 member...
SLC23A2	9962	SLC23A2	solute carrier family 23 member...
SLC29A1	2030	SLC29A1	solute carrier family 29 member...
SLC7A6	9057	SLC7A6	solute carrier family 7 member ...
SLTM	79811	SLTM	SAFB like transcription modulat...
SMC2	10592	SMC2	structural maintenance of chrom...
SMC4	10051	SMC4	structural maintenance of chrom...
SOCS2	8835	SOCS2	suppressor of cytokine signalin...
SPATA5	166378	SPATA5	spermatogenesis associated 5 [S...
SPOP	8405	SPOP	speckle type BTB/POZ protein [S...
SQRDL	58472	SQOR	sulfide quinone oxidoreductase ...
SSX2IP	117178	SSX2IP	SSX family member 2 interacting...
STRN3	29966	STRN3	striatin 3 [Source:HGNC Symbol;...
SYT4	6860	SYT4	synaptotagmin 4 [Source:HGNC Sy...
TARDBP	23435	TARDBP	TAR DNA binding protein [Source...
TCF1	6927	HNF1A	HNF1 homeobox A [Source:HGNC Sy...
TEAD3	7005	TEAD3	TEA domain transcription factor...
THOP1	7064	THOP1	thimet oligopeptidase 1 [Source...

TK1	7083	TK1	thymidine kinase 1 [Source:HGNC...
TM9SF3	56889	TM9SF3	transmembrane 9 superfamily mem...
TMOD3	29766	TMOD3	tropomodulin 3 [Source:HGNC Sym...
TNFSF10	8743	TNFSF10	TNF superfamily member 10 [Sour...
TNFSF11	8600	TNFSF11	TNF superfamily member 11 [Sour...
TOP2A	7153	TOP2A	DNA topoisomerase II alpha [Sou...
TOP2B	7155	TOP2B	DNA topoisomerase II beta [Sour...
TRAP1A			
TRIP13	9319	TRIP13	thyroid hormone receptor intera...
Timm8a1	1678	TIMM8A	translocase of inner mitochondr...
UFD1L	7353	UFD1	ubiquitin recognition factor in...
UNG	7374	UNG	uracil DNA glycosylase [Source:...
USP39	10713	USP39	ubiquitin specific peptidase 39...
USP52	9924	PAN2	poly(A) specific ribonuclease s...
VPS4B	9525	VPS4B	vacuolar protein sorting 4 homo...
XPO1	7514	XPO1	exportin 1 [Source:HGNC Symbol;...
YTHDF3	253943	YTHDF3	YTH N6-methyladenosine RNA bind...
YWHAE	7531	YWHAE	tyrosine 3-monooxygenase/trypto...
ZFP281			
ZFP644			
ZFX	7543	ZFX	zinc finger protein X-linked [S...
ZKSCAN1	7586	ZKSCAN1	zinc finger with KRAB and SCAN ...
ZMYM6	9204	ZMYM6	zinc finger MYM-type containing...
ZNF638	27332	ZNF638	zinc finger protein 638 [Source..
